# Supplementary material for: Evaluating the Use of ABBA–BABA Statistics to Locate Introgressed Loci
Source: Mol Biol Evol. 2014 Sep 22;32(1):244–57. doi: 10.1093/molbev/msu269 (PMC4271521; doi:10.1093/molbev/msu269)
Supplement: Supplementary Data [file supp_32_1_244__index.html]

Evaluating the use of ABBA-BABA statistics to locate introgressed loci — Evaluating the Use of ABBA–BABA Statistics to Locate Introgressed Loci — Evaluating the Use of ABBA–BABA Statistics to Locate Introgressed Loci — Supplementary Data 

# Evaluating the Use of ABBA–BABA Statistics to Locate Introgressed Loci

## Supplementary Data

files

**Files in this Data Supplement:**

- Supplementary Data - pdf file
