# Supplementary material for: Evaluating the Use of ABBA–BABA Statistics to Locate Introgressed Loci
Source: Mol Biol Evol. 2014 Sep 22;32(1):244–57. doi: 10.1093/molbev/msu269 (PMC4271521; doi:10.1093/molbev/msu269)
Supplement: Supplementary Data [file supp_msu269_Martin_Davey_Jiggins_Supplementary_Information.pdf]

Supplementary Information

Evaluating the use of ABBA-BABA statistics to locate introgressed loci

Simon H. Martin, John W. Davey, Chris D. Jiggins

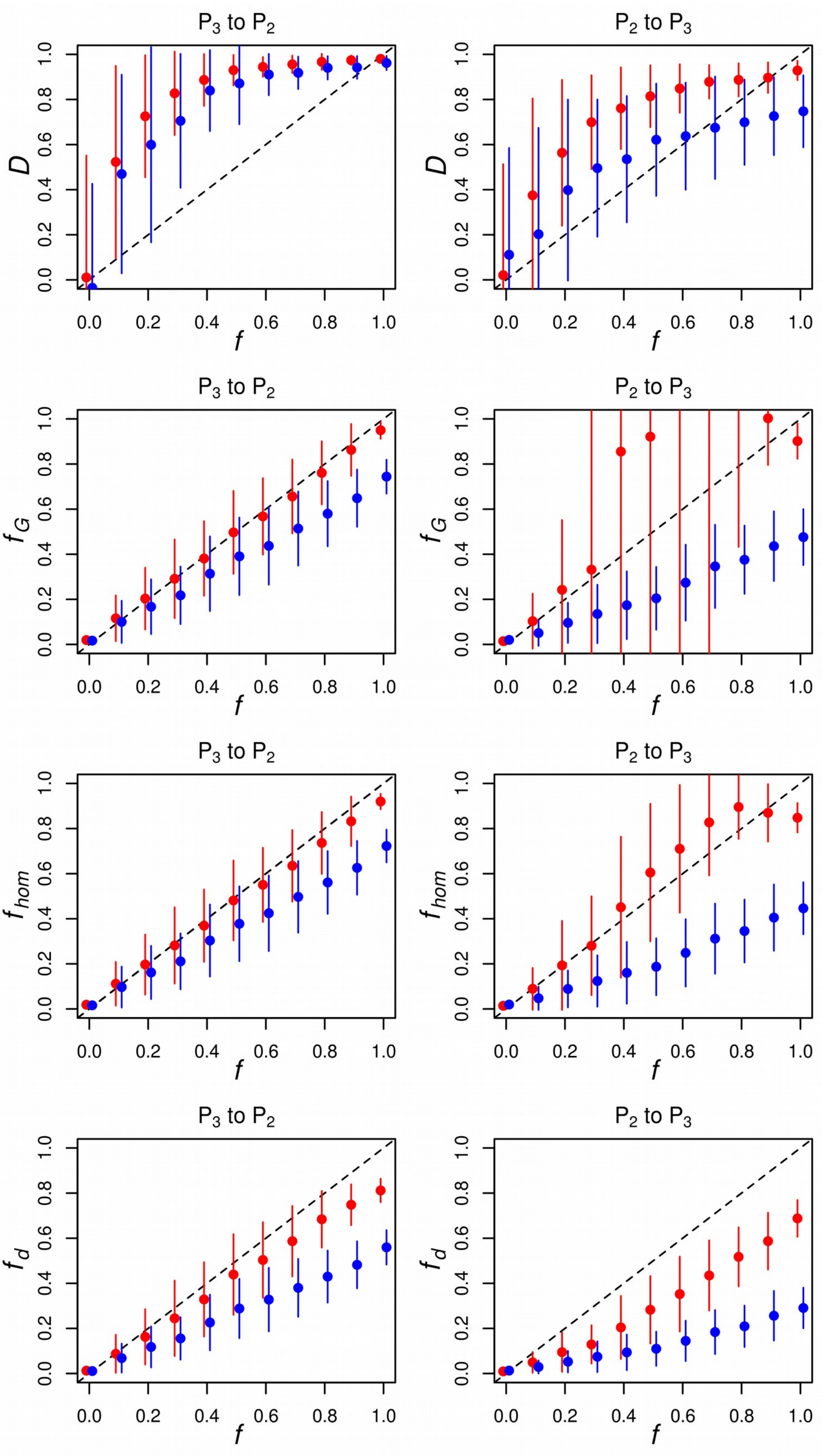

Figure S1 Comparing statistics to detect and quantify introgression (A) window = 5 kb, 4Nr = 0.01 Plots show means and standard deviations for *D* and *f* estimators, calculated over 100 simulated sequences (See Methods for details). Simulations covered 11 different values of *f*, the proportion of introgression. Gene flow was simulated either from P_3 to P_2 (conventional model, left-hand column) or from P_2 to P_3 (right-hand

column). Dashed diagonal lines show the expectation of a perfect estimator of *f*. Two times of gene flow are shown: 0.1 (red) and 0.5 (blue) × 4N generations ago.

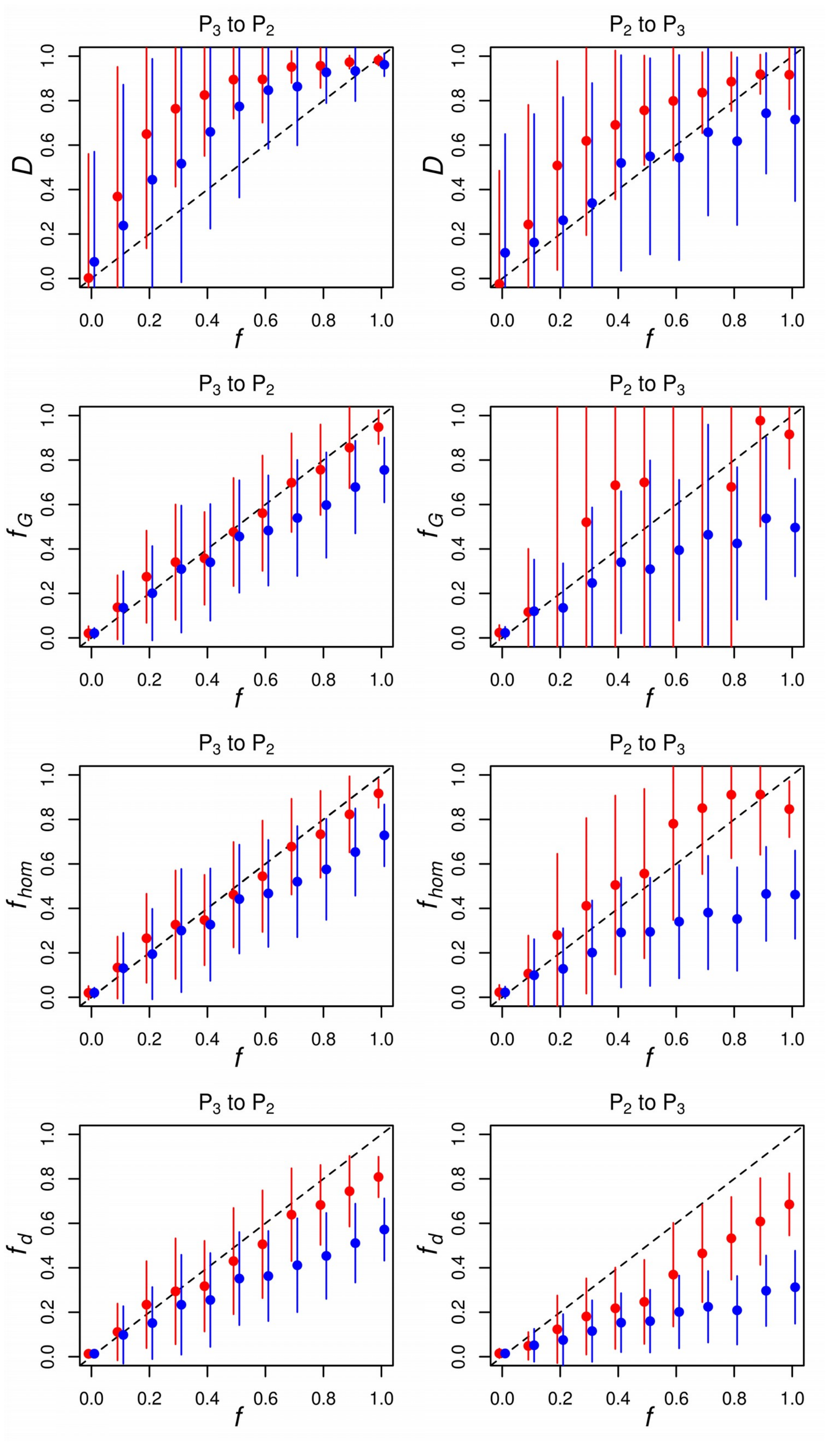

Figure S1 Comparing statistics to detect and quantify introgression (B) window = 5 kb, 4Nr = 0.001

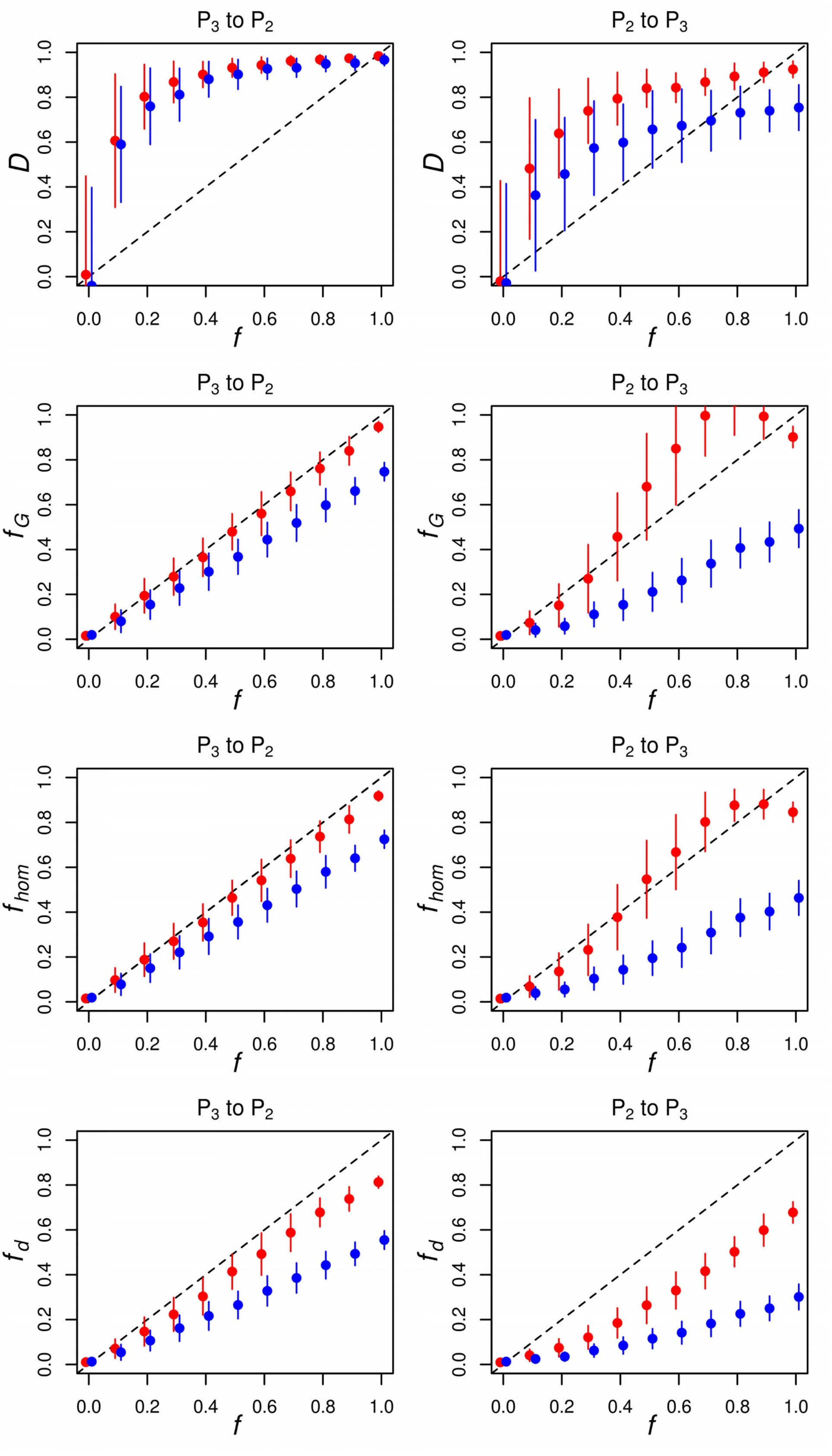

Figure S1 Comparing statistics to detect and quantify introgression (C) window = 5 kb, 4Nr = 0.1

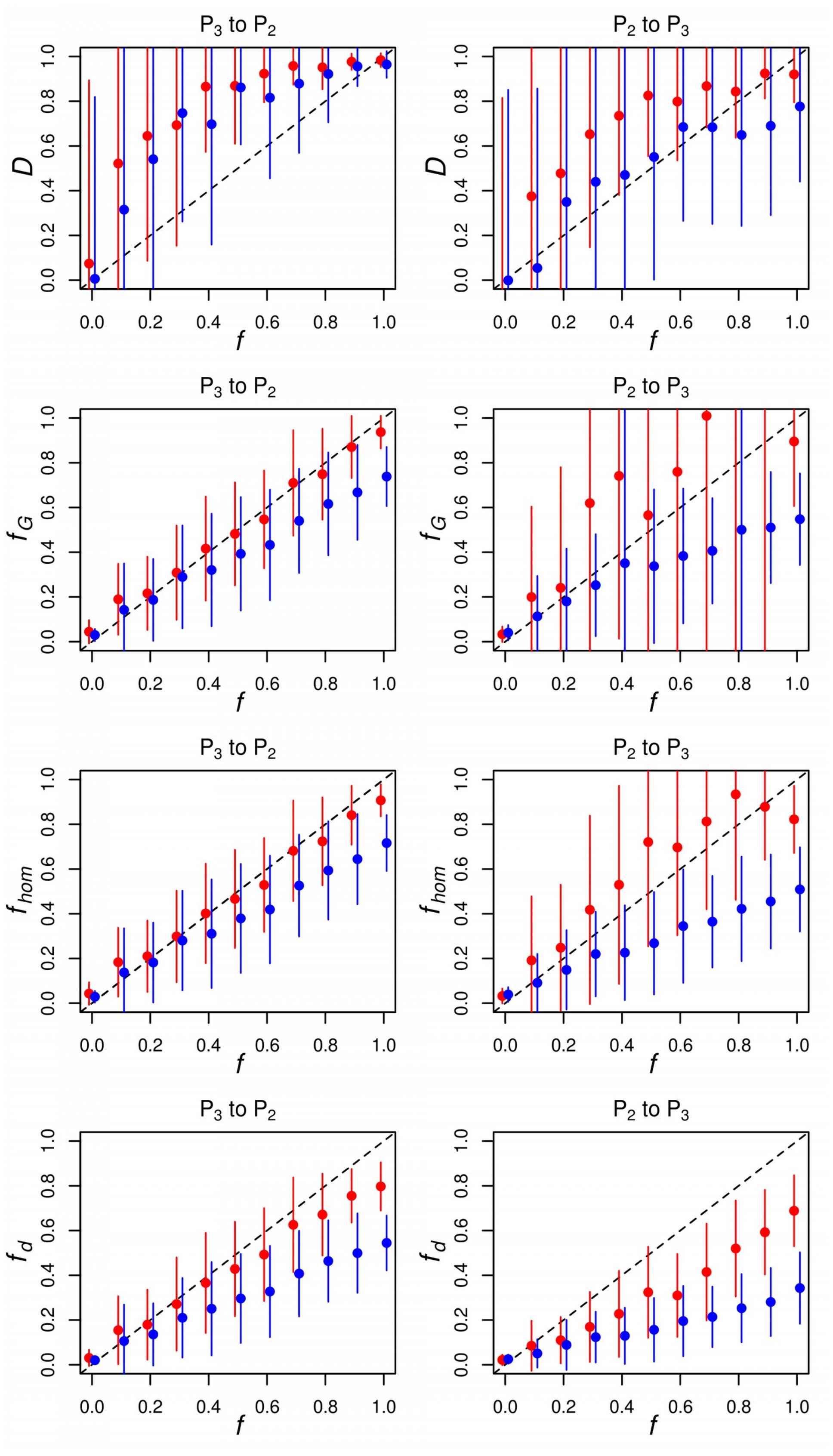

Figure S1 Comparing statistics to detect and quantify introgression (D) window = 1 kb, 4Nr = 0.01

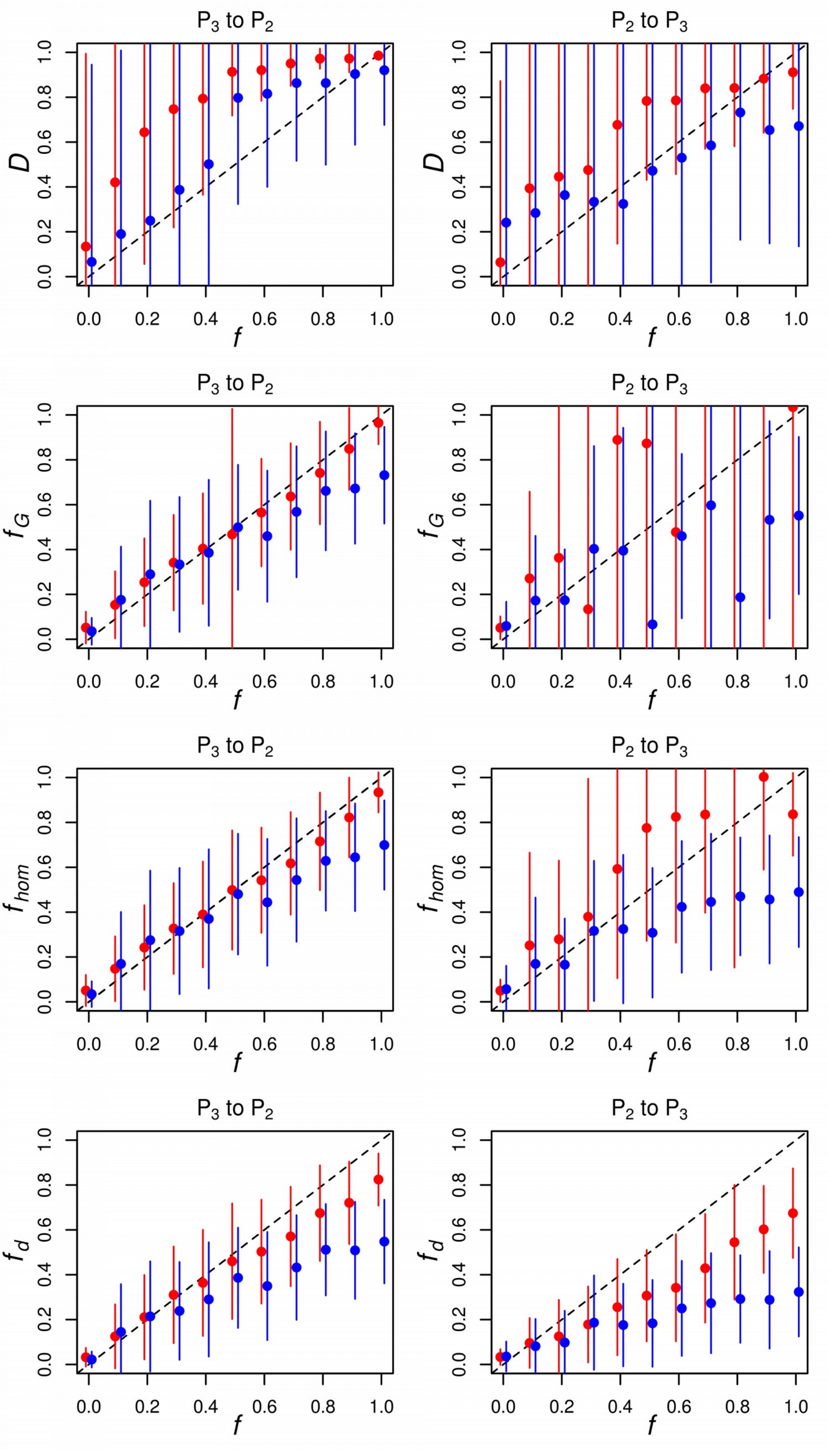

Figure S1 Comparing statistics to detect and quantify introgression (E) window = 1 kb, 4Nr = 0.001

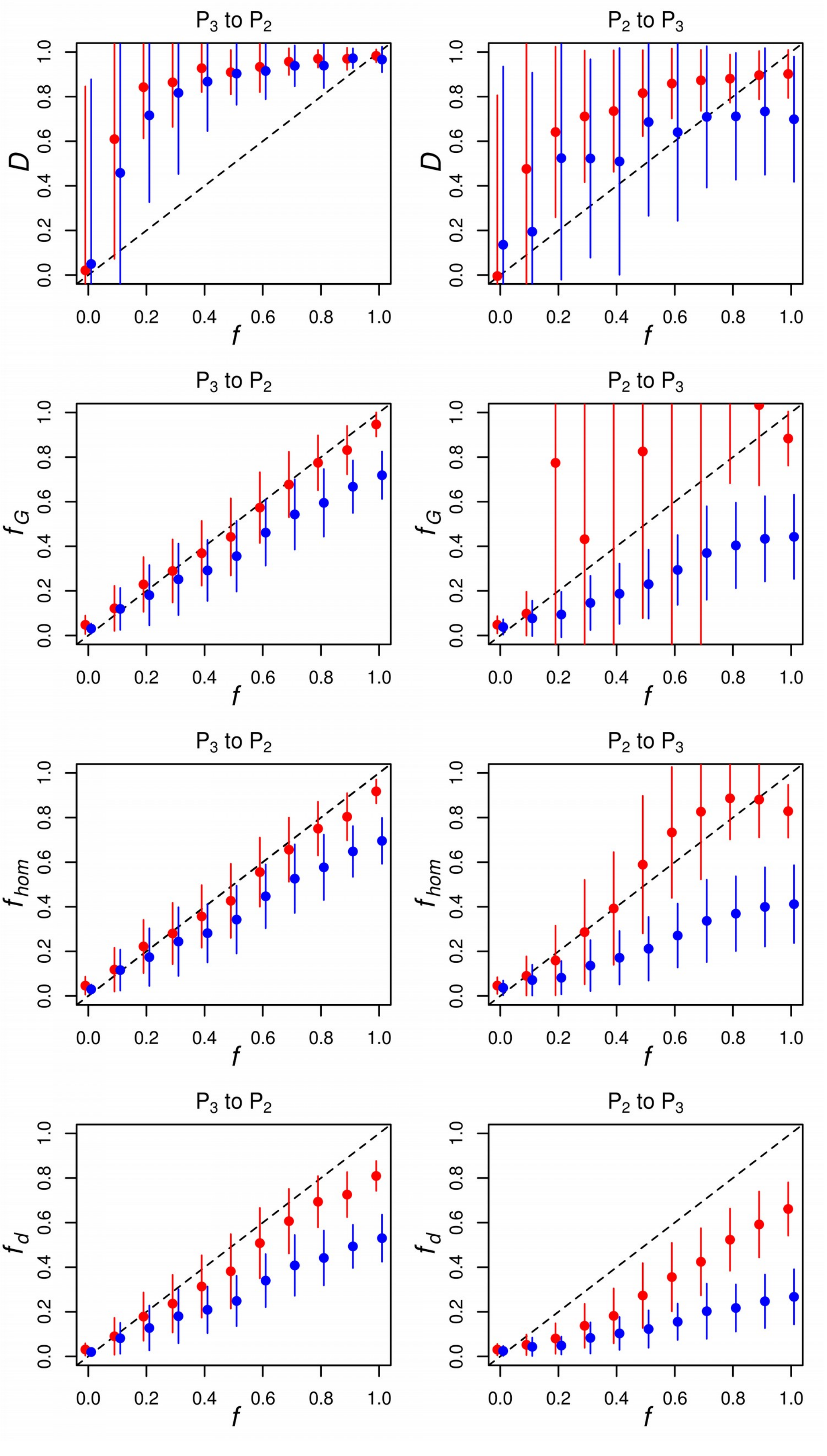

Figure S1 Comparing statistics to detect and quantify introgression (F) window = 1 kb, 4Nr = 0.1

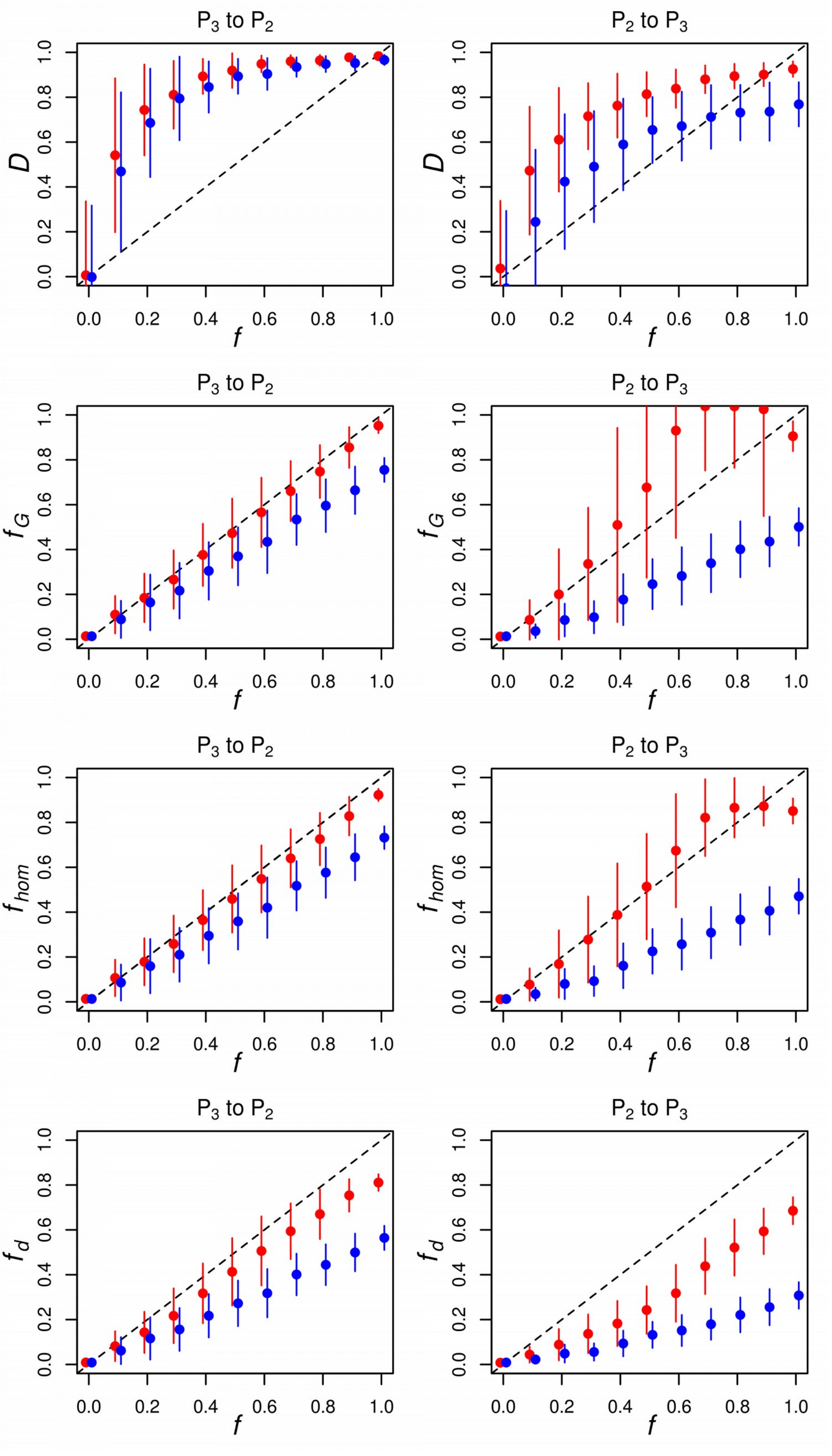

Figure S1 Comparing statistics to detect and quantify introgression (G) window = 10 kb, 4Nr = 0.01

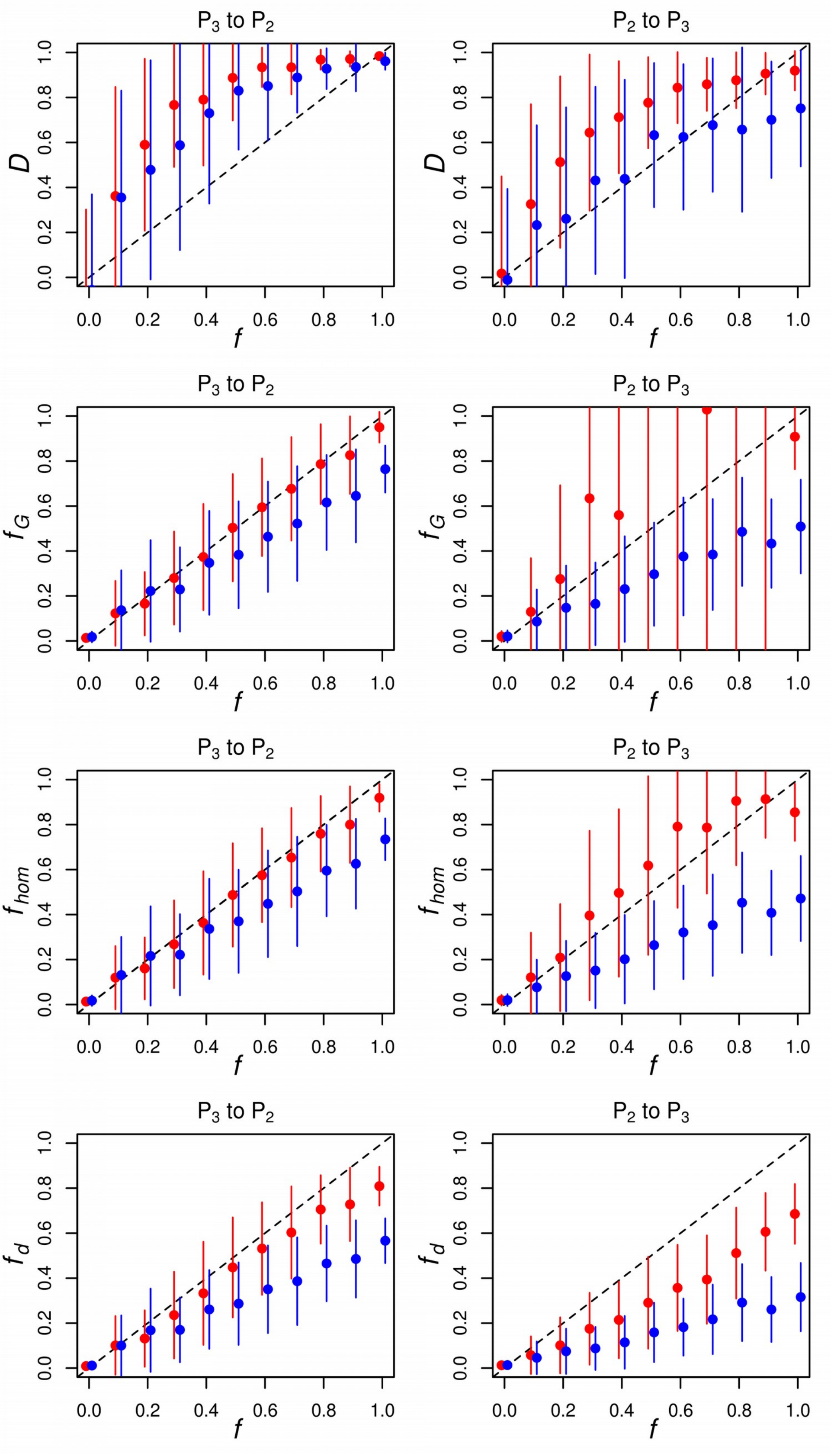

Figure S1 Comparing statistics to detect and quantify introgression (H) window = 10 kb, 4Nr = 0.001

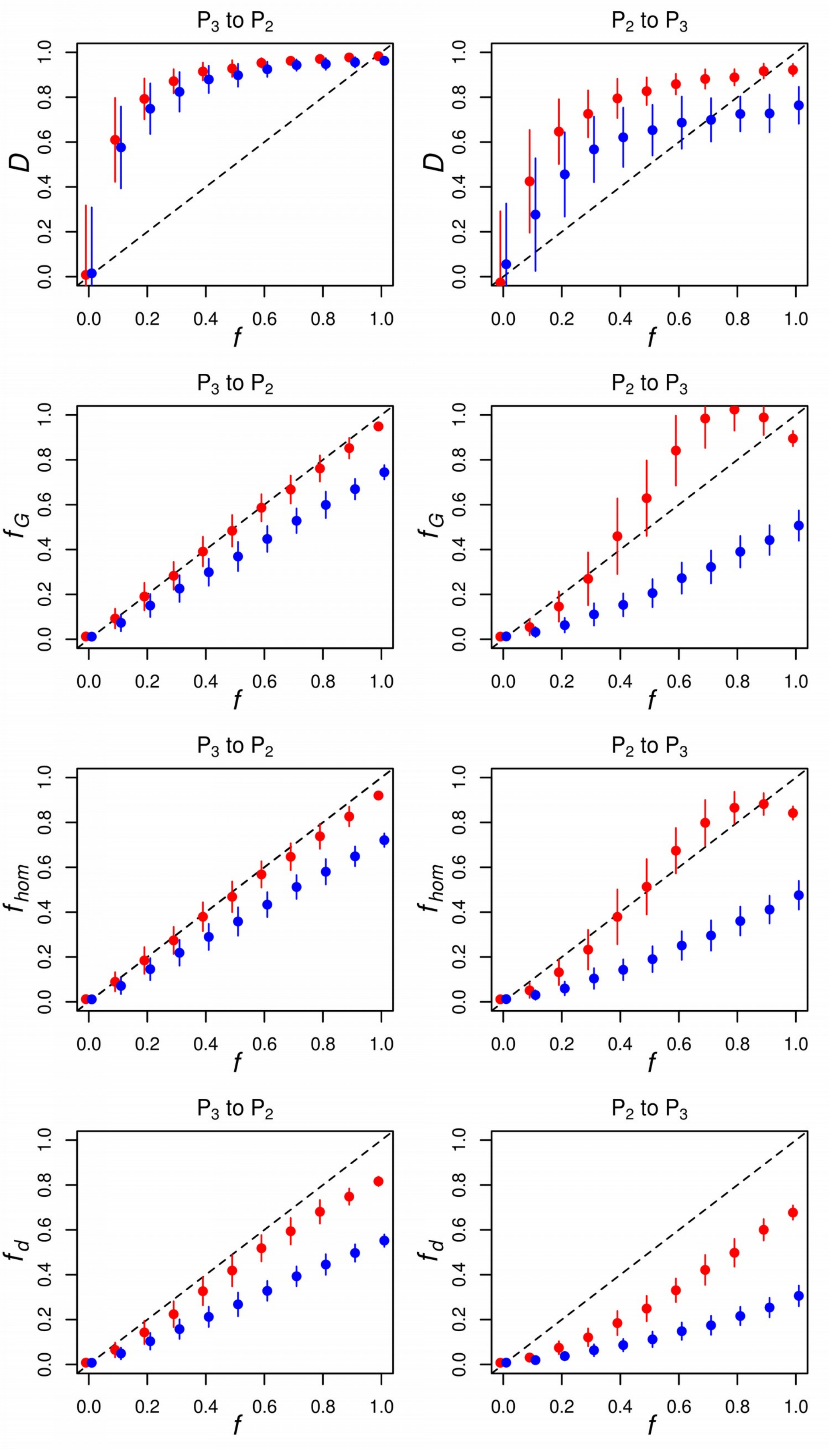

Figure S1 Comparing statistics to detect and quantify introgression (I) window = 10 kb, 4Nr = 0.1

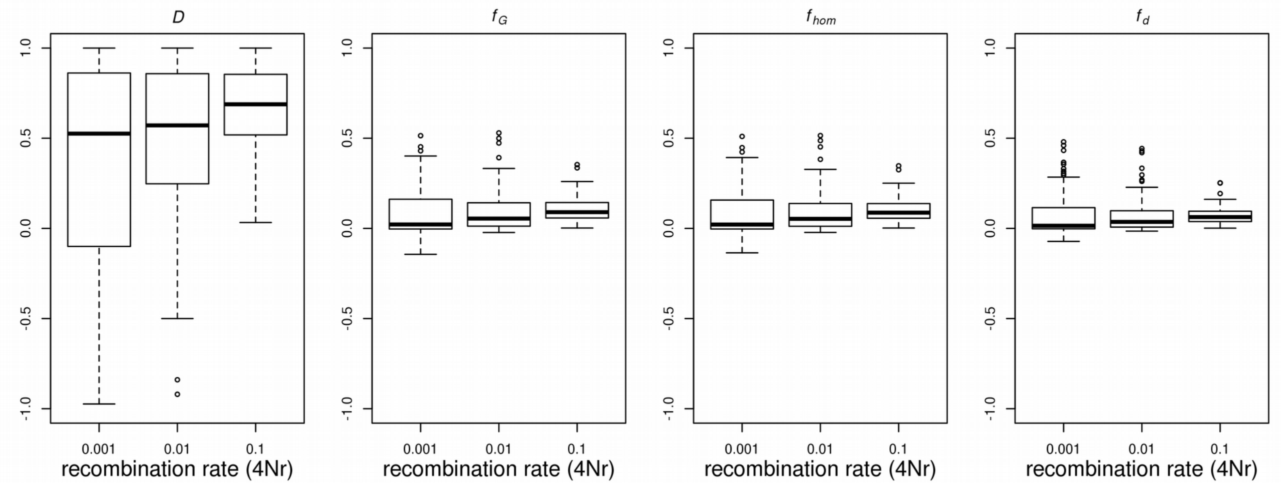

Figure S2. Effects of recombination rate one the *D* statistic and *f* estimators. Boxplots show the distribution of values for 100 simulated 5 kb windows, with *f* = 0.1, three different values for the population recombination rate (4Nr) were tested: 0.001, 0.01 and 0.1.

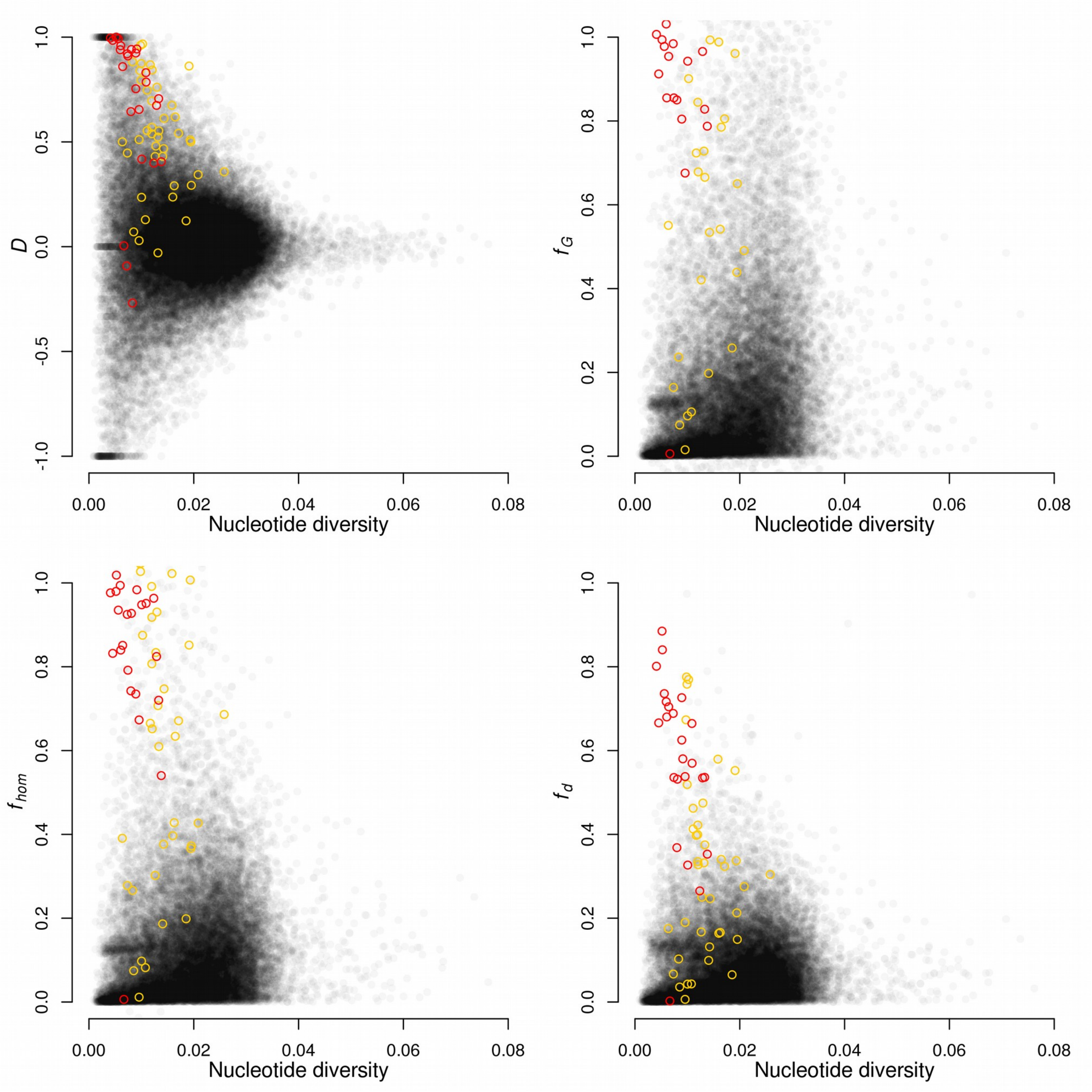

Figure S3. Effects of genetic diversity on the *D* and *f* statistics in *Heliconius* whole genome data. **A,B.** Values of *D* and the three *f* statistics for non-overlapping 5 kb windows across the genome, plotted against nucleotide diversity. *f* values are only plotted for windows with D≥0. Data from Martin et al. 2013. Taxa used are as follows, P_1: *Heliconius melpomene aglaope*, P_2: *Heliconius melpomene amaryllis*, P_3: *Heliconius timareta thelxinoe*, O: four *Heliconius* species from the silvaniform clade. Colored points show windows located within the wing patterning loci *HmB* (red) and *HmYb* (yellow), see Methods.

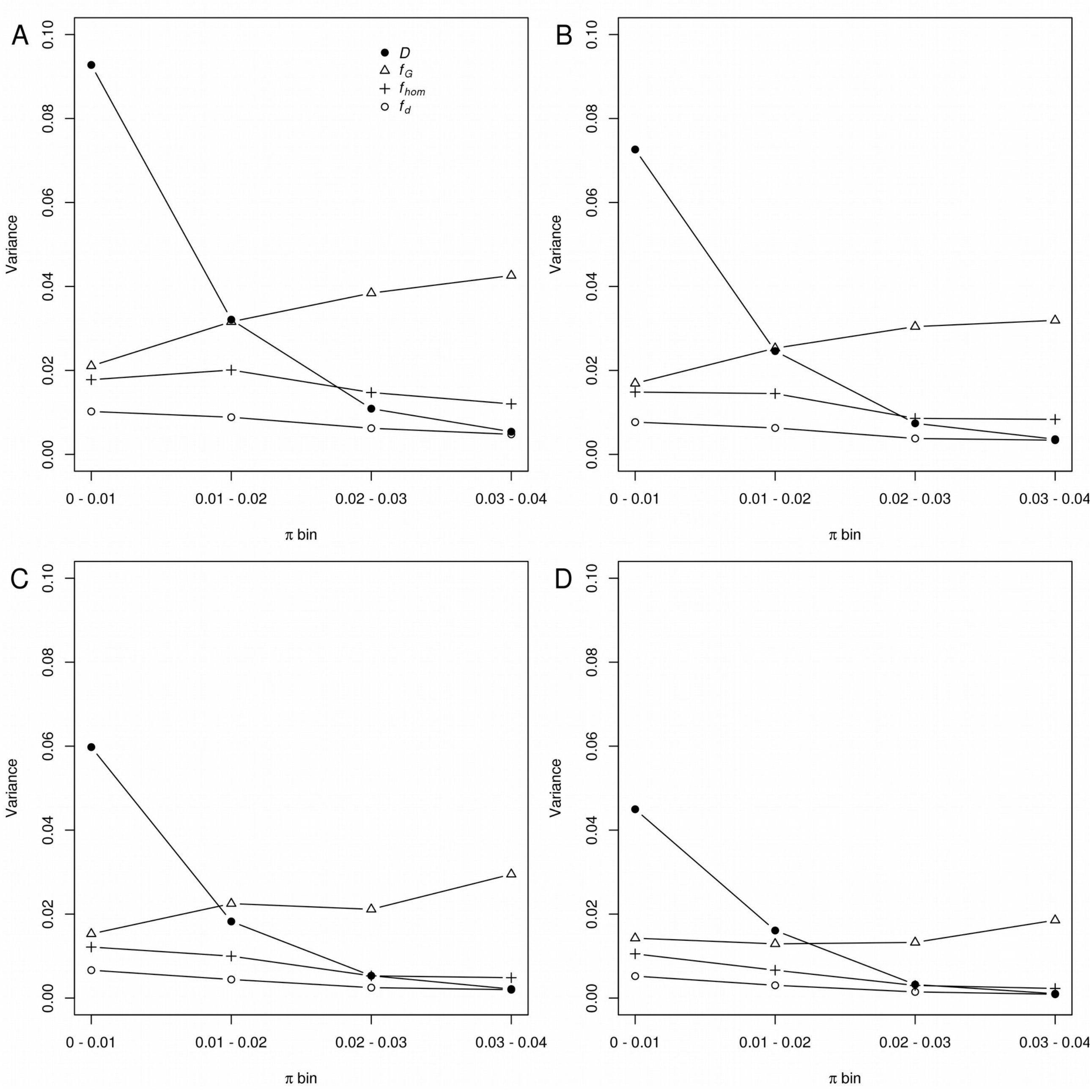

Figure S4. Variance in *D* and *f* statistics plotted in bins of nucleotide diversity

Each statistic was binned into one of four bins according to average nucleotide diversity (π) in the three populations. Variance was calculated for each bin. For all four statistics, only windows with D ≥ 0 were considered because *f* statistics are meaningless when *D* is negative, and can range from 0 to negative infinity. For f_G and f_{hom}, windows with values greater than one were excluded for calculating variance. Four window sizes were examined: 5 kb (**A**), 10 kb (**B**), 20 kb (**C**) and 50 kb (**D**).

Table S1. d_{XY} between P_2 and P_3 for different sets of windows, for models with GENE FLOW FROM P_3 to P_2.

The first three columns give the split times for the model and the time of gene flow in units of 4N generations ago (see Fig. 4A). The 'Alt' and 'Bg' columns give d_{XY} between P_2 and P_3 for the 1000 Alternate and 9000 Background windows, respectively. For each of the introgression statistics, there are three columns giving d_{XY} between P_2 and P_3 for outliers (windows with the top 10% of values), non-outliers (the remaining 90% of windows) and then the percentage of the outliers that corresponded to real Alternate windows in the simulation (i.e. the level of accuracy of the statistic in identifying gene flow windows). Highlighted blocks indicate cases where d_{XY} in the Alternate or outlier windows was significantly lower than in the Background or non-outlier windows.

					D			f_d			f_G			f_{hom}		
t_{23}	t_{12}	t_{GF}	Alt	Bg	outlier	non-outlier	% cor.	outlier	non-outlier	% cor.	outlier	non-outlier	% cor.	outlier	non-outlier	% cor.
0.6	0.4	0.2	0.014	0.022	0.015	0.021	74.1	0.014	0.022	95.8	0.014	0.022	96.4	0.014	0.022	96.5
0.8	0.4	0.2	0.014	0.025	0.015	0.025	83.3	0.014	0.025	99.8	0.014	0.025	99.7	0.014	0.025	99.7
0.8	0.6	0.2	0.014	0.025	0.015	0.025	84.6	0.014	0.025	99.4	0.014	0.025	99.2	0.014	0.025	99.3
0.8	0.6	0.4	0.018	0.025	0.019	0.025	69.3	0.018	0.025	91.2	0.018	0.025	91.7	0.018	0.025	91.8
1	0.4	0.2	0.014	0.029	0.016	0.029	83.7	0.014	0.029	100	0.014	0.029	100	0.014	0.029	100
1	0.6	0.2	0.014	0.029	0.015	0.029	87	0.014	0.029	99.9	0.014	0.029	99.9	0.014	0.029	99.9
1	0.6	0.4	0.018	0.029	0.020	0.029	76.6	0.018	0.029	98.9	0.018	0.029	99.1	0.018	0.029	99.1
1	0.8	0.2	0.014	0.029	0.015	0.029	90.5	0.014	0.029	99.9	0.014	0.029	99.9	0.014	0.029	99.9
1	0.8	0.4	0.018	0.029	0.019	0.029	81.9	0.018	0.029	98.5	0.018	0.029	98.6	0.018	0.029	98.5
1	0.8	0.6	0.022	0.029	0.023	0.029	64.9	0.022	0.029	88.5	0.022	0.029	88.9	0.022	0.029	88.9
1.2	0.4	0.2	0.014	0.033	0.016	0.033	84	0.014	0.033	100	0.014	0.033	100	0.014	0.033	100
1.2	0.6	0.2	0.014	0.033	0.016	0.033	86.6	0.014	0.033	100	0.014	0.033	100	0.014	0.033	100
1.2	0.6	0.4	0.018	0.033	0.020	0.033	79.1	0.018	0.033	100	0.018	0.033	100	0.018	0.033	100
1.2	0.8	0.2	0.014	0.033	0.015	0.033	91.1	0.014	0.033	100	0.014	0.033	100	0.014	0.033	100
1.2	0.8	0.4	0.018	0.033	0.020	0.033	83.7	0.018	0.033	99.9	0.018	0.033	99.9	0.018	0.033	99.9
1.2	0.8	0.6	0.022	0.033	0.024	0.033	74.1	0.022	0.033	98.5	0.022	0.033	98.7	0.022	0.033	98.9
1.2	1	0.2	0.014	0.033	0.015	0.033	94	0.014	0.033	100	0.014	0.033	100	0.014	0.033	100
1.2	1	0.4	0.018	0.033	0.019	0.033	88.6	0.018	0.033	99.9	0.018	0.033	99.8	0.018	0.033	99.8
1.2	1	0.6	0.022	0.033	0.023	0.033	79.8	0.022	0.033	97.8	0.022	0.033	97.9	0.022	0.033	97.8
1.2	1	0.8	0.026	0.033	0.028	0.033	61.6	0.026	0.033	87.2	0.026	0.033	87.2	0.026	0.033	87.4
1.4	0.4	0.2	0.014	0.037	0.017	0.037	81.8	0.014	0.037	100	0.014	0.037	100	0.014	0.037	100
1.4	0.6	0.2	0.014	0.037	0.017	0.037	84.9	0.014	0.037	100	0.014	0.037	100	0.014	0.037	100
1.4	0.6	0.4	0.018	0.037	0.021	0.037	80.3	0.018	0.037	100	0.018	0.037	100	0.018	0.037	100
1.4	0.8	0.2	0.014	0.037	0.017	0.037	86.2	0.014	0.037	100	0.014	0.037	100	0.014	0.037	100
1.4	0.8	0.4	0.018	0.037	0.021	0.037	81.8	0.018	0.037	100	0.018	0.037	100	0.018	0.037	100
1.4	0.8	0.6	0.021	0.037	0.025	0.037	75.2	0.021	0.037	100	0.021	0.037	100	0.021	0.037	100
1.4	1	0.2	0.014	0.037	0.015	0.037	91.4	0.014	0.037	100	0.014	0.037	100	0.014	0.037	100
1.4	1	0.4	0.018	0.037	0.020	0.037	87.8	0.018	0.037	100	0.018	0.037	100	0.018	0.037	100
1.4	1	0.6	0.021	0.037	0.024	0.037	80.8	0.021	0.037	99.8	0.021	0.037	99.8	0.021	0.037	99.8
1.4	1	0.8	0.025	0.037	0.028	0.037	69.7	0.026	0.037	97.6	0.026	0.037	97.8	0.026	0.037	97.8
1.4	1.2	0.2	0.014	0.037	0.015	0.037	94.7	0.014	0.037	100	0.014	0.037	100	0.014	0.037	100
1.4	1.2	0.4	0.018	0.037	0.019	0.037	91.2	0.018	0.037	100	0.018	0.037	100	0.018	0.037	100
1.4	1.2	0.6	0.021	0.037	0.023	0.037	85.7	0.021	0.037	99.8	0.021	0.037	99.7	0.021	0.037	99.8
1.4	1.2	0.8	0.025	0.037	0.027	0.037	76.5	0.026	0.037	97.2	0.026	0.037	97.2	0.026	0.037	97.2
1.4	1.2	1	0.029	0.037	0.031	0.037	59.4	0.030	0.037	85	0.030	0.037	86.2	0.030	0.037	86.3

1.6	0.4	0.2	0.014	0.041	0.019	0.040	78.9	0.014	0.041	100	0.014	0.041	100	0.014	0.041	100
1.6	0.6	0.2	0.014	0.041	0.018	0.040	82.7	0.014	0.041	100	0.014	0.041	100	0.014	0.041	100
1.6	0.6	0.4	0.018	0.041	0.022	0.040	79.4	0.018	0.041	100	0.018	0.041	100	0.018	0.041	100
1.6	0.8	0.2	0.014	0.041	0.017	0.040	86.8	0.014	0.041	100	0.014	0.041	100	0.014	0.041	100
1.6	0.8	0.4	0.018	0.041	0.021	0.040	83.6	0.018	0.041	100	0.018	0.041	100	0.018	0.041	100
1.6	0.8	0.6	0.022	0.041	0.025	0.040	78.3	0.022	0.041	100	0.022	0.041	100	0.022	0.041	100
1.6	1	0.2	0.014	0.041	0.016	0.040	89.4	0.014	0.041	100	0.014	0.041	100	0.014	0.041	100
1.6	1	0.4	0.018	0.041	0.021	0.040	85.9	0.018	0.041	100	0.018	0.041	100	0.018	0.041	100
1.6	1	0.6	0.022	0.041	0.025	0.040	81.1	0.022	0.041	100	0.022	0.041	100	0.022	0.041	100
1.6	1	0.8	0.025	0.041	0.029	0.040	75.1	0.025	0.041	99.9	0.025	0.041	99.9	0.025	0.041	99.9
1.6	1.2	0.2	0.014	0.041	0.015	0.041	94.2	0.014	0.041	100	0.014	0.041	100	0.014	0.041	100
1.6	1.2	0.4	0.018	0.041	0.019	0.041	91.4	0.018	0.041	100	0.018	0.041	100	0.018	0.041	100
1.6	1.2	0.6	0.022	0.041	0.023	0.041	88	0.022	0.041	100	0.022	0.041	100	0.022	0.041	100
1.6	1.2	0.8	0.025	0.041	0.028	0.041	81.2	0.025	0.041	99.9	0.025	0.041	99.8	0.025	0.041	99.8
1.6	1.2	1	0.029	0.041	0.032	0.041	71.1	0.029	0.041	98	0.029	0.041	98.2	0.029	0.041	98.2
1.6	1.4	0.2	0.014	0.041	0.015	0.041	96.4	0.014	0.041	100	0.014	0.041	100	0.014	0.041	100
1.6	1.4	0.4	0.018	0.041	0.019	0.041	94.9	0.018	0.041	100	0.018	0.041	100	0.018	0.041	100
1.6	1.4	0.6	0.022	0.041	0.023	0.041	91.7	0.022	0.041	99.9	0.022	0.041	99.9	0.022	0.041	99.9
1.6	1.4	0.8	0.025	0.041	0.027	0.041	86	0.025	0.041	99.5	0.025	0.041	99.3	0.025	0.041	99.4
1.6	1.4	1.2	0.033	0.041	0.035	0.041	60.2	0.033	0.041	85.4	0.033	0.041	85.7	0.033	0.041	86.2
1.6	1.4	1	0.029	0.041	0.031	0.041	76.3	0.029	0.041	97	0.029	0.041	96.5	0.029	0.041	96.6
1.8	0.4	0.2	0.014	0.045	0.022	0.044	73.8	0.014	0.045	100	0.014	0.045	100	0.014	0.045	100
1.8	0.6	0.2	0.014	0.045	0.020	0.044	78.7	0.014	0.045	100	0.014	0.045	100	0.014	0.045	100
1.8	0.6	0.4	0.018	0.045	0.023	0.044	76.9	0.018	0.045	100	0.018	0.045	100	0.018	0.045	100
1.8	0.8	0.2	0.014	0.045	0.018	0.044	84.5	0.014	0.045	100	0.014	0.045	100	0.014	0.045	100
1.8	0.8	0.4	0.018	0.045	0.022	0.044	81.8	0.018	0.045	100	0.018	0.045	100	0.018	0.045	100
1.8	0.8	0.6	0.022	0.045	0.026	0.044	77.5	0.022	0.045	100	0.022	0.045	100	0.022	0.045	100
1.8	1	0.2	0.014	0.044	0.017	0.044	88.6	0.014	0.044	100	0.014	0.044	100	0.014	0.044	100
1.8	1	0.4	0.018	0.044	0.021	0.044	86.1	0.018	0.044	100	0.018	0.044	100	0.018	0.044	100
1.8	1	0.6	0.022	0.044	0.025	0.044	81.8	0.022	0.044	100	0.022	0.044	100	0.022	0.044	100
1.8	1	0.8	0.026	0.044	0.029	0.044	77	0.026	0.044	100	0.026	0.044	100	0.026	0.044	100
1.8	1.2	0.2	0.014	0.045	0.016	0.044	91.6	0.014	0.045	100	0.014	0.045	100	0.014	0.045	100
1.8	1.2	0.4	0.018	0.045	0.020	0.044	89.6	0.018	0.045	100	0.018	0.045	100	0.018	0.045	100
1.8	1.2	0.6	0.022	0.045	0.024	0.044	85.3	0.022	0.045	100	0.022	0.045	100	0.022	0.045	100
1.8	1.2	0.8	0.026	0.045	0.029	0.044	80.4	0.026	0.045	100	0.026	0.045	100	0.026	0.045	100
1.8	1.2	1	0.029	0.045	0.033	0.044	74.5	0.029	0.045	99.8	0.029	0.045	99.8	0.029	0.045	99.8
1.8	1.4	0.2	0.014	0.045	0.015	0.044	94.1	0.014	0.045	100	0.014	0.045	100	0.014	0.045	100
1.8	1.4	0.4	0.018	0.045	0.019	0.044	92.7	0.018	0.045	100	0.018	0.045	100	0.018	0.045	100
1.8	1.4	0.6	0.022	0.045	0.024	0.044	89.9	0.022	0.045	100	0.022	0.045	100	0.022	0.045	100
1.8	1.4	0.8	0.026	0.045	0.028	0.044	87	0.026	0.045	100	0.026	0.045	100	0.026	0.045	100
1.8	1.4	1.2	0.033	0.045	0.036	0.044	71.6	0.033	0.045	97.4	0.033	0.045	97.6	0.033	0.045	97.6
1.8	1.4	1	0.029	0.045	0.032	0.044	82.3	0.029	0.045	99.8	0.029	0.045	99.8	0.029	0.045	99.8
1.8	1.6	0.2	0.014	0.045	0.015	0.044	97.3	0.014	0.045	100	0.014	0.045	100	0.014	0.045	100
1.8	1.6	0.4	0.018	0.045	0.018	0.044	96.3	0.018	0.045	100	0.018	0.045	100	0.018	0.045	100
1.8	1.6	0.6	0.022	0.045	0.023	0.044	93.9	0.022	0.045	100	0.022	0.045	100	0.022	0.045	100
1.8	1.6	0.8	0.026	0.045	0.027	0.044	90	0.026	0.045	100	0.026	0.045	100	0.026	0.045	100
1.8	1.6	1.2	0.033	0.045	0.035	0.044	74.1	0.034	0.045	95.9	0.034	0.045	95.9	0.033	0.045	95.9
1.8	1.6	1.4	0.037	0.045	0.039	0.044	55.5	0.037	0.044	81.1	0.037	0.044	81.8	0.037	0.044	81.9
1.8	1.6	1	0.029	0.045	0.031	0.044	86.2	0.029	0.045	99.5	0.029	0.045	99.5	0.029	0.045	99.5
2	0.4	0.2	0.014	0.048	0.023	0.047	71.6	0.014	0.048	100	0.014	0.048	100	0.014	0.048	100
2	0.6	0.2	0.014	0.048	0.022	0.047	74.2	0.014	0.048	100	0.014	0.048	100	0.014	0.048	100

2	0.6	0.4	0.018	0.048	0.026	0.047	72.8	0.018	0.048	100	0.018	0.048	100	0.018	0.048	100
2	0.8	0.2	0.014	0.048	0.020	0.048	82.3	0.014	0.048	100	0.014	0.048	100	0.014	0.048	100
2	0.8	0.4	0.018	0.048	0.023	0.048	80.3	0.018	0.048	100	0.018	0.048	100	0.018	0.048	100
2	0.8	0.6	0.022	0.048	0.027	0.048	77.5	0.022	0.048	100	0.022	0.048	100	0.022	0.048	100
2	1	0.2	0.014	0.048	0.018	0.048	86.1	0.014	0.048	100	0.014	0.048	100	0.014	0.048	100
2	1	0.4	0.018	0.048	0.022	0.048	83.6	0.018	0.048	100	0.018	0.048	100	0.018	0.048	100
2	1	0.6	0.022	0.048	0.026	0.048	81.4	0.022	0.048	100	0.022	0.048	100	0.022	0.048	100
2	1	0.8	0.025	0.048	0.030	0.048	77.4	0.025	0.048	100	0.025	0.048	100	0.025	0.048	100
2	1.2	0.2	0.014	0.048	0.017	0.048	89.1	0.014	0.048	100	0.014	0.048	100	0.014	0.048	100
2	1.2	0.4	0.018	0.048	0.021	0.048	87.4	0.018	0.048	100	0.018	0.048	100	0.018	0.048	100
2	1.2	0.6	0.022	0.048	0.025	0.048	85.7	0.022	0.048	100	0.022	0.048	100	0.022	0.048	100
2	1.2	0.8	0.025	0.048	0.029	0.048	82.4	0.025	0.048	100	0.025	0.048	100	0.025	0.048	100
2	1.2	1	0.029	0.048	0.033	0.048	77.4	0.029	0.048	100	0.029	0.048	100	0.029	0.048	100
2	1.4	0.2	0.014	0.048	0.016	0.048	93.3	0.014	0.048	100	0.014	0.048	100	0.014	0.048	100
2	1.4	0.4	0.018	0.048	0.020	0.048	91.8	0.018	0.048	100	0.018	0.048	100	0.018	0.048	100
2	1.4	0.6	0.022	0.048	0.024	0.048	90.3	0.022	0.048	100	0.022	0.048	100	0.022	0.048	100
2	1.4	0.8	0.025	0.048	0.028	0.048	86.8	0.025	0.048	100	0.025	0.048	100	0.025	0.048	100
2	1.4	1.2	0.033	0.048	0.036	0.048	75.6	0.033	0.048	99.6	0.033	0.048	99.6	0.033	0.048	99.6
2	1.4	1	0.029	0.048	0.032	0.048	82.2	0.029	0.048	100	0.029	0.048	100	0.029	0.048	100
2	1.6	0.2	0.014	0.048	0.015	0.048	95.6	0.014	0.048	100	0.014	0.048	100	0.014	0.048	100
2	1.6	0.4	0.018	0.048	0.019	0.048	94	0.018	0.048	100	0.018	0.048	100	0.018	0.048	100
2	1.6	0.6	0.022	0.048	0.023	0.048	92.9	0.022	0.048	100	0.022	0.048	100	0.022	0.048	100
2	1.6	0.8	0.025	0.048	0.027	0.048	89.2	0.025	0.048	100	0.025	0.048	100	0.025	0.048	100
2	1.6	1.2	0.033	0.048	0.035	0.048	80.2	0.033	0.048	99.2	0.033	0.048	99.3	0.033	0.048	99.3
2	1.6	1.4	0.037	0.048	0.039	0.048	70.6	0.037	0.048	97.9	0.037	0.048	97.8	0.037	0.048	97.8
2	1.6	1	0.029	0.048	0.032	0.048	85.5	0.029	0.048	99.8	0.029	0.048	99.9	0.029	0.048	99.9
2	1.8	0.2	0.014	0.048	0.015	0.048	97.7	0.014	0.048	100	0.014	0.048	100	0.014	0.048	100
2	1.8	0.4	0.018	0.048	0.019	0.048	96.3	0.018	0.048	100	0.018	0.048	100	0.018	0.048	100
2	1.8	0.6	0.022	0.048	0.023	0.048	95.4	0.022	0.048	100	0.022	0.048	100	0.022	0.048	100
2	1.8	0.8	0.025	0.048	0.027	0.048	93.4	0.025	0.048	100	0.025	0.048	100	0.025	0.048	100
2	1.8	1.2	0.033	0.048	0.035	0.048	84	0.033	0.048	99	0.033	0.048	99.1	0.033	0.048	99.1
2	1.8	1.4	0.037	0.048	0.039	0.048	74.6	0.037	0.048	96.6	0.037	0.048	96.7	0.037	0.048	96.6
2	1.8	1.6	0.041	0.048	0.043	0.048	57	0.041	0.048	81.5	0.041	0.048	82.7	0.041	0.048	82.7
2	1.8	1	0.029	0.048	0.031	0.048	89.4	0.029	0.048	99.9	0.029	0.048	99.9	0.029	0.048	99.9

Table S2. d_{XY} between P_2 and P_3 for different sets of windows, for models with GENE FLOW FROM P_2 to P_3.

The first three columns give the split times for the model and the time of gene flow in units of 4N generations ago (see Fig. 4B). The 'Alt' and 'Bg' columns give d_{XY} between P_2 and P_3 for the 1000 Alternate and 9000 Background windows, respectively. For each of the introgression statistics, there are three columns giving d_{XY} between P_2 and P_3 for outliers (windows with the top 10% of values), non-outliers (the remaining 90% of windows) and then the percentage of the outliers that corresponded to real Alternate windows in the simulation (i.e. the level of accuracy of the statistic in identifying gene flow windows). Highlighted blocks indicate cases where d_{XY} in the Alternate or outlier windows was significantly lower than in the Background or non-outlier windows.

					D			f_d			f_G			f_{hom}		
t_{23}	t_{12}	t_{GF}	Alt	Bg	outlier	non-outlier	% cor.	outlier	non-outlier	% cor.	outlier	non-outlier	% cor.	outlier	non-outlier	% cor.
0.6	0.4	0.2	0.014	0.022	0.017	0.021	45.6	0.015	0.022	80.4	0.014	0.022	83.5	0.014	0.022	82.5
0.8	0.4	0.2	0.014	0.025	0.020	0.025	41.3	0.015	0.025	87	0.015	0.025	90.2	0.015	0.025	89.2
0.8	0.6	0.2	0.014	0.025	0.016	0.025	69.9	0.014	0.025	97.5	0.014	0.025	98	0.014	0.025	97.8
0.8	0.6	0.4	0.018	0.025	0.021	0.025	38.1	0.019	0.025	71	0.019	0.025	73.6	0.019	0.025	73.3
1	0.4	0.2	0.014	0.029	0.023	0.028	31.8	0.015	0.029	90.7	0.015	0.029	93	0.015	0.029	92.6
1	0.6	0.2	0.014	0.029	0.019	0.029	59.9	0.014	0.029	98.7	0.014	0.029	99	0.014	0.029	99
1	0.6	0.4	0.018	0.029	0.025	0.029	31	0.020	0.029	77.2	0.019	0.029	80.3	0.019	0.029	79.2
1	0.8	0.2	0.014	0.029	0.016	0.029	82.8	0.014	0.029	99.7	0.014	0.029	99.7	0.014	0.029	99.7
1	0.8	0.4	0.018	0.029	0.021	0.029	67	0.018	0.029	92.6	0.018	0.029	93.3	0.018	0.029	92.9
1	0.8	0.6	0.021	0.029	0.025	0.029	36.4	0.023	0.029	67.7	0.023	0.029	69.8	0.023	0.029	69.4
1.2	0.4	0.2	0.014	0.033	0.028	0.032	25.1	0.015	0.033	93.3	0.015	0.033	94.8	0.015	0.033	94.3
1.2	0.6	0.2	0.014	0.033	0.022	0.032	54	0.014	0.033	99.4	0.014	0.033	99.6	0.014	0.033	99.5
1.2	0.6	0.4	0.018	0.033	0.028	0.032	26	0.020	0.033	82.4	0.019	0.033	85.3	0.020	0.033	84.5
1.2	0.8	0.2	0.014	0.033	0.018	0.033	76.7	0.014	0.033	99.8	0.014	0.033	99.8	0.014	0.033	99.8
1.2	0.8	0.4	0.018	0.033	0.023	0.033	58.2	0.018	0.033	96.2	0.018	0.033	96.6	0.018	0.033	96.4
1.2	0.8	0.6	0.021	0.033	0.028	0.032	29.7	0.024	0.033	74.2	0.023	0.033	77	0.023	0.033	76.7
1.2	1	0.2	0.014	0.033	0.016	0.033	89.8	0.014	0.033	99.9	0.014	0.033	99.9	0.014	0.033	99.9
1.2	1	0.4	0.018	0.033	0.020	0.033	80.9	0.018	0.033	99.1	0.018	0.033	99.1	0.018	0.033	99.1
1.2	1	0.6	0.022	0.033	0.025	0.033	64.1	0.022	0.033	91.2	0.022	0.033	91.9	0.022	0.033	91.8
1.2	1	0.8	0.026	0.033	0.029	0.033	35.6	0.027	0.033	62.6	0.027	0.033	63.8	0.027	0.033	63.5
1.4	0.4	0.2	0.014	0.037	0.032	0.035	20.3	0.015	0.037	94.4	0.015	0.037	95.7	0.015	0.037	95.5
1.4	0.6	0.2	0.014	0.037	0.025	0.036	48.5	0.014	0.037	99.6	0.014	0.037	99.7	0.014	0.037	99.6
1.4	0.6	0.4	0.018	0.037	0.032	0.035	22.5	0.020	0.037	85.8	0.020	0.037	88.7	0.020	0.037	88.2
1.4	0.8	0.2	0.014	0.037	0.021	0.036	67.6	0.014	0.037	99.9	0.014	0.037	100	0.014	0.037	100
1.4	0.8	0.4	0.018	0.037	0.026	0.036	50.8	0.018	0.037	97.4	0.018	0.037	97.9	0.018	0.037	97.7
1.4	0.8	0.6	0.021	0.037	0.032	0.036	24.7	0.024	0.037	78.8	0.024	0.037	81.5	0.024	0.037	80.9
1.4	1	0.2	0.014	0.037	0.017	0.037	83.1	0.014	0.037	100	0.014	0.037	100	0.014	0.037	100
1.4	1	0.4	0.018	0.037	0.022	0.036	72.2	0.018	0.037	99.2	0.018	0.037	99.4	0.018	0.037	99.3
1.4	1	0.6	0.022	0.037	0.027	0.036	56.4	0.022	0.037	94.4	0.022	0.037	95.1	0.022	0.037	95.1
1.4	1	0.8	0.026	0.037	0.033	0.036	28.6	0.028	0.037	68.6	0.028	0.037	71.5	0.028	0.037	71.4
1.4	1.2	0.2	0.014	0.037	0.015	0.037	92.6	0.014	0.037	100	0.014	0.037	100	0.014	0.037	100
1.4	1.2	0.4	0.018	0.037	0.020	0.037	87	0.018	0.037	99.9	0.018	0.037	99.9	0.018	0.037	99.9
1.4	1.2	0.6	0.022	0.037	0.024	0.037	77.2	0.022	0.037	98.3	0.022	0.037	98.4	0.022	0.037	98.3
1.4	1.2	0.8	0.026	0.037	0.029	0.037	59.4	0.027	0.037	88.3	0.026	0.037	88.6	0.027	0.037	88.5

1.4	1.2	1	0.029	0.037	0.033	0.037	31.4	0.031	0.037	57.6	0.031	0.037	59.4	0.031	0.037	59.2
1.6	0.4	0.2	0.014	0.041	0.035	0.038	16.6	0.015	0.041	95	0.015	0.041	96	0.015	0.041	95.9
1.6	0.6	0.2	0.014	0.041	0.029	0.039	40.7	0.014	0.041	99.7	0.014	0.041	99.9	0.014	0.041	99.9
1.6	0.6	0.4	0.018	0.041	0.036	0.039	16.8	0.020	0.041	87.6	0.020	0.041	89.9	0.020	0.041	89.4
1.6	0.8	0.2	0.014	0.041	0.023	0.040	64.1	0.014	0.041	99.9	0.014	0.041	99.9	0.014	0.041	99.9
1.6	0.8	0.4	0.018	0.041	0.029	0.040	46.5	0.018	0.041	98.3	0.018	0.041	98.9	0.018	0.041	98.8
1.6	0.8	0.6	0.021	0.041	0.036	0.039	21	0.024	0.040	82.4	0.024	0.041	85	0.024	0.041	84.6
1.6	1	0.2	0.014	0.041	0.019	0.040	78.3	0.014	0.041	100	0.014	0.041	100	0.014	0.041	100
1.6	1	0.4	0.018	0.041	0.025	0.040	67.3	0.018	0.041	99.7	0.018	0.041	99.8	0.018	0.041	99.8
1.6	1	0.6	0.022	0.041	0.030	0.040	50.6	0.022	0.041	96.5	0.022	0.041	96.8	0.022	0.041	96.7
1.6	1	0.8	0.026	0.041	0.036	0.040	25.6	0.029	0.040	74.6	0.028	0.040	76.9	0.028	0.040	76.7
1.6	1.2	0.2	0.014	0.041	0.016	0.041	90.2	0.014	0.041	100	0.014	0.041	100	0.014	0.041	100
1.6	1.2	0.4	0.018	0.041	0.021	0.040	82.7	0.018	0.041	100	0.018	0.041	100	0.018	0.041	100
1.6	1.2	0.6	0.022	0.041	0.026	0.040	73.4	0.022	0.041	99.3	0.022	0.041	99.3	0.022	0.041	99.2
1.6	1.2	0.8	0.026	0.041	0.032	0.040	54.8	0.027	0.041	92.1	0.026	0.041	92.8	0.027	0.041	92.6
1.6	1.2	1	0.029	0.041	0.037	0.040	27.1	0.032	0.040	66.8	0.032	0.041	69.6	0.032	0.041	68.9
1.6	1.4	0.2	0.014	0.041	0.015	0.041	95.4	0.014	0.041	100	0.014	0.041	100	0.014	0.041	100
1.6	1.4	0.4	0.018	0.041	0.019	0.041	92.6	0.018	0.041	100	0.018	0.041	100	0.018	0.041	100
1.6	1.4	0.6	0.021	0.041	0.024	0.041	86.3	0.021	0.041	99.7	0.021	0.041	99.7	0.021	0.041	99.8
1.6	1.4	0.8	0.025	0.041	0.028	0.040	77.9	0.026	0.041	97.7	0.026	0.041	97.7	0.026	0.041	97.6
1.6	1.4	1.2	0.033	0.041	0.037	0.040	35.6	0.035	0.041	58.1	0.035	0.041	59.2	0.035	0.041	59.1
1.6	1.4	1	0.029	0.041	0.033	0.040	61.1	0.030	0.041	88	0.030	0.041	88.5	0.030	0.041	88.1
1.8	0.4	0.2	0.014	0.045	0.040	0.042	13.4	0.015	0.044	95.7	0.015	0.044	96.5	0.015	0.044	96.4
1.8	0.6	0.2	0.014	0.045	0.032	0.043	37.2	0.014	0.045	99.8	0.014	0.045	100	0.014	0.045	100
1.8	0.6	0.4	0.018	0.045	0.040	0.042	15.5	0.020	0.044	89.3	0.020	0.044	90.7	0.020	0.044	90.5
1.8	0.8	0.2	0.014	0.045	0.026	0.043	58.1	0.014	0.045	100	0.014	0.045	100	0.014	0.045	100
1.8	0.8	0.4	0.018	0.045	0.033	0.043	41.3	0.018	0.044	99	0.018	0.044	99.1	0.018	0.044	99.1
1.8	0.8	0.6	0.021	0.045	0.040	0.043	18	0.024	0.044	85.2	0.024	0.044	87.1	0.024	0.044	86.7
1.8	1	0.2	0.014	0.044	0.021	0.044	73.9	0.014	0.044	100	0.014	0.044	100	0.014	0.044	100
1.8	1	0.4	0.018	0.044	0.027	0.043	63.3	0.018	0.044	99.9	0.018	0.044	99.9	0.018	0.044	99.9
1.8	1	0.6	0.022	0.044	0.033	0.043	45.8	0.022	0.044	97.3	0.022	0.044	97.9	0.022	0.044	97.7
1.8	1	0.8	0.026	0.044	0.040	0.043	21.6	0.029	0.044	78.4	0.029	0.044	80.9	0.029	0.044	80.6
1.8	1.2	0.2	0.014	0.045	0.018	0.044	84.4	0.014	0.045	100	0.014	0.045	100	0.014	0.045	100
1.8	1.2	0.4	0.018	0.045	0.023	0.044	77	0.018	0.045	100	0.018	0.045	100	0.018	0.045	100
1.8	1.2	0.6	0.022	0.045	0.028	0.044	67.2	0.022	0.045	99.5	0.022	0.045	99.5	0.022	0.045	99.5
1.8	1.2	0.8	0.026	0.045	0.034	0.044	49.3	0.027	0.045	94.2	0.026	0.045	95.2	0.026	0.045	95.2
1.8	1.2	1	0.029	0.045	0.040	0.043	24.6	0.033	0.044	72.6	0.032	0.044	75.6	0.032	0.044	75.1
1.8	1.4	0.2	0.014	0.045	0.016	0.044	91.6	0.014	0.045	100	0.014	0.045	100	0.014	0.045	100
1.8	1.4	0.4	0.018	0.045	0.020	0.044	89	0.018	0.045	100	0.018	0.045	100	0.018	0.045	100
1.8	1.4	0.6	0.021	0.045	0.025	0.044	83.6	0.021	0.045	100	0.021	0.045	100	0.021	0.045	100
1.8	1.4	0.8	0.025	0.045	0.030	0.044	74.5	0.026	0.045	98.8	0.026	0.045	99	0.026	0.045	98.9
1.8	1.4	1.2	0.033	0.045	0.040	0.044	32	0.036	0.044	66.5	0.036	0.044	68.8	0.036	0.044	68.1
1.8	1.4	1	0.029	0.045	0.035	0.044	57.1	0.030	0.045	92.8	0.030	0.045	93.5	0.030	0.045	93.3
1.8	1.6	0.2	0.014	0.045	0.015	0.044	96.5	0.014	0.045	100	0.014	0.045	100	0.014	0.045	100
1.8	1.6	0.4	0.018	0.045	0.019	0.044	95.2	0.018	0.045	100	0.018	0.045	100	0.018	0.045	100
1.8	1.6	0.6	0.022	0.045	0.023	0.044	91.2	0.022	0.045	100	0.022	0.045	100	0.022	0.045	100
1.8	1.6	0.8	0.025	0.045	0.027	0.044	86.2	0.025	0.045	99.7	0.025	0.045	99.7	0.025	0.045	99.8
1.8	1.6	1.2	0.033	0.045	0.036	0.044	58.1	0.034	0.044	87.6	0.034	0.044	87.5	0.034	0.044	87.3
1.8	1.6	1.4	0.037	0.045	0.040	0.044	35.5	0.039	0.044	58.6	0.039	0.044	59.3	0.039	0.044	59.2
1.8	1.6	1	0.029	0.045	0.032	0.044	76.2	0.029	0.045	97.3	0.029	0.045	97.5	0.029	0.045	97.5
2	0.4	0.2	0.014	0.048	0.044	0.045	11.9	0.015	0.048	95.9	0.015	0.048	96.6	0.015	0.048	96.5

2	0.6	0.2	0.014	0.048	0.036	0.046	34.5	0.014	0.048	99.9	0.014	0.048	100	0.014	0.048	100
2	0.6	0.4	0.018	0.048	0.044	0.046	13.6	0.020	0.048	90.2	0.020	0.048	91.6	0.020	0.048	91.2
2	0.8	0.2	0.014	0.048	0.029	0.047	54.9	0.014	0.048	100	0.014	0.048	100	0.014	0.048	100
2	0.8	0.4	0.018	0.048	0.036	0.046	37.2	0.018	0.048	99	0.018	0.048	99.2	0.018	0.048	99.1
2	0.8	0.6	0.021	0.048	0.044	0.046	14.9	0.025	0.048	86	0.024	0.048	88.3	0.024	0.048	87.9
2	1	0.2	0.014	0.048	0.024	0.047	70.3	0.014	0.048	100	0.014	0.048	100	0.014	0.048	100
2	1	0.4	0.018	0.048	0.029	0.047	59.3	0.018	0.048	99.9	0.018	0.048	99.9	0.018	0.048	99.9
2	1	0.6	0.022	0.048	0.036	0.047	41	0.022	0.048	97.6	0.022	0.048	98.1	0.022	0.048	98.1
2	1	0.8	0.026	0.048	0.043	0.046	18.7	0.029	0.048	80.7	0.029	0.048	82.5	0.029	0.048	82.2
2	1.2	0.2	0.014	0.048	0.019	0.048	82.4	0.014	0.048	100	0.014	0.048	100	0.014	0.048	100
2	1.2	0.4	0.018	0.048	0.025	0.048	73.8	0.018	0.048	100	0.018	0.048	100	0.018	0.048	100
2	1.2	0.6	0.022	0.048	0.030	0.047	63.9	0.022	0.048	99.6	0.022	0.048	99.6	0.022	0.048	99.6
2	1.2	0.8	0.026	0.048	0.037	0.047	45.2	0.026	0.048	95.9	0.026	0.048	96.3	0.026	0.048	96.2
2	1.2	1	0.029	0.048	0.044	0.047	21.2	0.033	0.048	76.4	0.033	0.048	78.3	0.033	0.048	78.2
2	1.4	0.2	0.014	0.048	0.017	0.048	89.6	0.014	0.048	100	0.014	0.048	100	0.014	0.048	100
2	1.4	0.4	0.018	0.048	0.022	0.048	85.3	0.018	0.048	100	0.018	0.048	100	0.018	0.048	100
2	1.4	0.6	0.021	0.048	0.027	0.048	78.3	0.021	0.048	100	0.021	0.048	100	0.021	0.048	100
2	1.4	0.8	0.025	0.048	0.032	0.048	67.5	0.026	0.048	99.4	0.026	0.048	99.4	0.026	0.048	99.4
2	1.4	1.2	0.033	0.048	0.043	0.047	29.3	0.037	0.048	71.2	0.036	0.048	73.3	0.036	0.048	73.2
2	1.4	1	0.029	0.048	0.037	0.047	52.1	0.030	0.048	94.5	0.030	0.048	94.9	0.030	0.048	94.9
2	1.6	0.2	0.014	0.048	0.016	0.048	94	0.014	0.048	100	0.014	0.048	100	0.014	0.048	100
2	1.6	0.4	0.018	0.048	0.020	0.048	91.5	0.018	0.048	100	0.018	0.048	100	0.018	0.048	100
2	1.6	0.6	0.022	0.048	0.025	0.048	86.9	0.022	0.048	100	0.022	0.048	100	0.022	0.048	100
2	1.6	0.8	0.025	0.048	0.029	0.048	82	0.025	0.048	99.7	0.025	0.048	99.8	0.025	0.048	99.8
2	1.6	1.2	0.033	0.048	0.039	0.048	56.2	0.034	0.048	91.5	0.034	0.048	92.1	0.034	0.048	92.1
2	1.6	1.4	0.037	0.048	0.043	0.048	33.5	0.040	0.048	66.5	0.039	0.048	68.2	0.039	0.048	67.7
2	1.6	1	0.029	0.048	0.034	0.048	71.8	0.029	0.048	98.5	0.029	0.048	98.7	0.029	0.048	98.7
2	1.8	0.2	0.014	0.048	0.015	0.048	97.2	0.014	0.048	100	0.014	0.048	100	0.014	0.048	100
2	1.8	0.4	0.018	0.048	0.019	0.048	95.9	0.018	0.048	100	0.018	0.048	100	0.018	0.048	100
2	1.8	0.6	0.022	0.048	0.023	0.048	93.7	0.022	0.048	100	0.022	0.048	100	0.022	0.048	100
2	1.8	0.8	0.026	0.048	0.027	0.048	89.6	0.026	0.048	100	0.026	0.048	100	0.026	0.048	100
2	1.8	1.2	0.033	0.048	0.036	0.048	74.7	0.034	0.048	96.7	0.034	0.048	96.5	0.034	0.048	96.5
2	1.8	1.4	0.037	0.048	0.041	0.048	56.7	0.038	0.048	83.2	0.038	0.048	83.5	0.038	0.048	83.3
2	1.8	1.6	0.041	0.048	0.044	0.048	33.7	0.043	0.048	56.3	0.043	0.048	57.1	0.043	0.048	57.2
2	1.8	1	0.029	0.048	0.032	0.048	85.4	0.029	0.048	99.7	0.029	0.048	99.5	0.029	0.048	99.5

Table S3. d_{XY} between P_2 and P_3 for different sets of windows, for models with ANCESTRAL STRUCTURE

The first three columns give the split times for the model in units of 4N generations ago (see Fig. 4C). The 'Alt' and 'Bg' columns give d_{XY} between P_2 and P_3 for the 1000 Alternate and 9000 Background windows, respectively. For each of the introgression statistics, there are three columns giving d_{XY} between P_2 and P_3 for outliers (windows with the top 10% of values), non-outliers (the remaining 90% of windows) and then the percentage of the outliers that corresponded to real Alternate windows in the simulation (i.e. the level of accuracy of the statistic in identifying structure windows). Highlighted blocks indicate cases where d_{XY} in the Alternate or outlier windows was significantly lower than in the Background or non-outlier windows.

					D			f_d			f_G			f_{hom}		
t_{23}	t_{12}	t_{STR}	Alt	Bg	outlier	non-outlier	% cor.	outlier	non-outlier	% cor.	outlier	non-outlier	% cor.	outlier	non-outlier	% cor.
0.4	0.2	0.6	0.018	0.018	**0.017**	0.018	52.8	**0.017**	0.018	58.9	**0.017**	0.018	61	**0.017**	0.018	63.4
0.4	0.2	0.8	0.018	0.018	**0.017**	0.018	80.2	**0.017**	0.018	82.9	**0.017**	0.018	82.7	**0.017**	0.018	85.6
0.4	0.2	1	0.018	0.018	**0.017**	0.018	92.4	**0.017**	0.018	93.5	**0.017**	0.018	92.3	**0.017**	0.018	94.7
0.4	0.2	1.2	0.018	0.018	0.018	0.018	97	0.018	0.018	97.9	0.017	0.018	95.5	0.018	0.018	97.9
0.4	0.2	1.4	0.018	0.018	0.018	0.018	98.9	0.018	0.018	99.2	0.018	0.018	97.2	0.018	0.018	99.3
0.4	0.2	1.6	0.018	0.018	0.018	0.018	99.5	0.018	0.018	99.7	0.018	0.018	98.4	0.018	0.018	99.6
0.4	0.2	1.8	0.018	0.018	0.018	0.018	99.8	0.018	0.018	100	0.018	0.018	98.9	0.018	0.018	99.8
0.4	0.2	2	0.018	0.018	0.018	0.018	99.8	0.018	0.018	99.9	0.018	0.018	99	0.018	0.018	99.8
0.6	0.2	0.8	0.021	0.022	**0.021**	0.022	47.2	**0.021**	0.022	65.6	**0.021**	0.022	70.9	**0.021**	0.022	71.2
0.6	0.2	1	0.022	0.022	**0.021**	0.022	73.2	**0.021**	0.022	86.7	**0.021**	0.022	90	**0.021**	0.022	90.9
0.6	0.2	1.2	0.022	0.022	**0.021**	0.022	87.8	0.021	0.022	96	0.021	0.022	96.9	0.021	0.022	97.2
0.6	0.2	1.4	0.021	0.022	**0.021**	0.022	93.6	0.021	0.022	98.5	0.021	0.022	98.6	0.021	0.022	99.1
0.6	0.2	1.6	0.022	0.022	0.021	0.022	96.6	0.022	0.022	99.4	0.022	0.022	99.6	0.022	0.022	99.6
0.6	0.2	1.8	0.022	0.022	0.021	0.022	98	0.022	0.022	99.7	0.022	0.022	99.8	0.022	0.022	99.9
0.6	0.2	2	0.022	0.022	0.022	0.022	98.6	0.022	0.022	99.9	0.022	0.022	100	0.022	0.022	100
0.6	0.4	0.8	0.021	0.022	**0.020**	0.022	44.1	**0.020**	0.022	55.8	**0.020**	0.022	59.3	**0.020**	0.022	59.7
0.6	0.4	1	0.022	0.022	**0.021**	0.022	70.8	**0.021**	0.022	80.4	**0.021**	0.022	80.9	**0.021**	0.022	82
0.6	0.4	1.2	0.022	0.022	**0.021**	0.022	86.1	**0.021**	0.022	92.3	**0.021**	0.022	92.7	**0.021**	0.022	93.3
0.6	0.4	1.4	0.021	0.022	**0.021**	0.022	92.5	**0.021**	0.022	96.9	**0.021**	0.022	96.6	**0.021**	0.022	97.3
0.6	0.4	1.6	0.022	0.022	0.021	0.022	96.1	0.022	0.022	98.8	0.021	0.022	98.3	0.022	0.022	98.8
0.6	0.4	1.8	0.022	0.022	0.021	0.022	97.5	0.021	0.022	99.4	0.021	0.022	99.1	0.021	0.022	99.4
0.6	0.4	2	0.022	0.022	0.022	0.022	98.4	0.022	0.022	99.8	0.022	0.022	99.3	0.022	0.022	99.6
0.8	0.2	1	0.026	0.025	**0.025**	0.026	39.2	**0.025**	0.026	65.9	**0.025**	0.026	72.2	**0.025**	0.026	72.6
0.8	0.2	1.2	0.026	0.025	**0.025**	0.026	64.3	0.025	0.026	88.1	0.025	0.026	91.5	0.025	0.026	91.6
0.8	0.2	1.4	0.025	0.025	**0.025**	0.026	80.8	0.025	0.026	96.8	0.025	0.026	97.8	0.025	0.026	98
0.8	0.2	1.6	0.025	0.025	**0.025**	0.026	87.3	0.025	0.026	98.7	0.025	0.025	99.5	0.025	0.025	99.5
0.8	0.2	1.8	0.026	0.025	0.025	0.026	91.7	0.026	0.025	99.6	0.026	0.025	99.7	0.026	0.025	99.7
0.8	0.2	2	0.025	0.025	**0.025**	0.026	94.3	0.025	0.025	99.6	0.025	0.025	99.7	0.025	0.025	99.7
0.8	0.4	1	0.026	0.025	**0.024**	0.026	38.7	**0.025**	0.026	59.7	**0.025**	0.026	63.7	**0.025**	0.026	63.6
0.8	0.4	1.2	0.026	0.025	**0.025**	0.026	64.5	**0.025**	0.026	84.4	**0.025**	0.026	86.5	0.025	0.026	87.4
0.8	0.4	1.4	0.025	0.025	**0.025**	0.026	81.1	0.025	0.025	95	0.025	0.025	96.1	0.025	0.025	96.4
0.8	0.4	1.6	0.025	0.025	**0.025**	0.026	87.5	0.025	0.025	98	0.025	0.025	98.2	0.025	0.025	98.4
0.8	0.4	1.8	0.026	0.025	0.025	0.025	91.8	0.026	0.025	99.5	0.025	0.025	99.3	0.026	0.025	99.6
0.8	0.4	2	0.025	0.025	**0.025**	0.025	95.1	0.025	0.025	99.8	0.025	0.025	99.7	0.025	0.025	99.7
0.8	0.6	1	0.026	0.025	**0.024**	0.026	38.5	**0.024**	0.026	52.5	**0.024**	0.026	53.9	**0.024**	0.026	54.7
0.8	0.6	1.2	0.026	0.025	**0.025**	0.026	64.9	**0.025**	0.026	80	**0.025**	0.026	80.1	**0.025**	0.026	81.7

0.8	0.6	1.4	0.025	0.025	**0.025**	0.026	81.9	**0.025**	0.026	92.3	**0.025**	0.026	92.5	**0.025**	0.026	93.3
0.8	0.6	1.6	0.025	0.025	**0.025**	0.026	89.7	**0.025**	0.026	96.8	**0.025**	0.026	96.4	**0.025**	0.026	97.1
0.8	0.6	1.8	0.026	0.025	0.025	0.026	94	0.025	0.026	98.9	0.025	0.026	98.8	0.025	0.026	99
0.8	0.6	2	0.025	0.025	**0.025**	0.026	96.3	0.025	0.026	99.6	0.025	0.026	99.1	0.025	0.026	99.3
1	0.2	1.2	0.029	0.029	**0.028**	0.029	29	**0.029**	0.029	68.1	**0.029**	0.029	75.5	**0.029**	0.029	75.5
1	0.2	1.4	0.029	0.029	**0.028**	0.029	57.7	**0.029**	0.029	91.4	0.029	0.029	93.9	0.029	0.029	94.1
1	0.2	1.6	0.029	0.029	**0.029**	0.029	72.7	0.029	0.029	97.3	0.029	0.029	98.5	0.029	0.029	98.7
1	0.2	1.8	0.029	0.029	**0.029**	0.029	82.2	0.029	0.029	99.3	0.029	0.029	99.8	0.029	0.029	99.8
1	0.2	2	0.029	0.029	**0.029**	0.029	86.8	0.029	0.029	99.8	0.029	0.029	99.9	0.029	0.029	99.9
1	0.4	1.2	0.029	0.029	**0.028**	0.029	28	**0.029**	0.029	63.6	**0.029**	0.029	69	**0.029**	0.029	69.3
1	0.4	1.4	0.029	0.029	**0.028**	0.029	55.3	**0.029**	0.029	87.9	0.029	0.029	91.8	0.029	0.029	92.2
1	0.4	1.6	0.029	0.029	**0.028**	0.029	70.8	0.029	0.029	96.4	0.029	0.029	97.4	0.029	0.029	97.6
1	0.4	1.8	0.029	0.029	**0.029**	0.029	79.9	0.029	0.029	98.7	0.029	0.029	99.5	0.029	0.029	99.5
1	0.4	2	0.029	0.029	**0.029**	0.029	85.7	0.029	0.029	99.6	0.029	0.029	99.7	0.029	0.029	99.9
1	0.6	1.2	0.029	0.029	**0.028**	0.029	28.8	**0.028**	0.029	55.8	**0.028**	0.029	59.7	**0.028**	0.029	60
1	0.6	1.4	0.029	0.029	**0.028**	0.029	57.3	**0.029**	0.029	82.1	**0.029**	0.029	85.8	**0.029**	0.029	86.4
1	0.6	1.6	0.029	0.029	**0.028**	0.029	73.2	**0.029**	0.029	94.5	0.029	0.029	95.5	0.029	0.029	95.8
1	0.6	1.8	0.029	0.029	**0.029**	0.029	82.9	0.029	0.029	97.9	0.029	0.029	98.4	0.029	0.029	98.5
1	0.6	2	0.029	0.029	**0.029**	0.029	88.6	0.029	0.029	99.2	0.029	0.029	99.2	0.029	0.029	99.3
1	0.8	1.2	0.029	0.029	**0.028**	0.030	32.7	**0.028**	0.029	49.9	**0.028**	0.029	51.9	**0.028**	0.029	52.6
1	0.8	1.4	0.029	0.029	**0.028**	0.029	61.8	**0.028**	0.029	78.1	**0.028**	0.029	79	**0.028**	0.029	79.7
1	0.8	1.6	0.029	0.029	**0.029**	0.029	78.5	**0.029**	0.029	92.1	**0.029**	0.029	91.6	**0.029**	0.029	92
1	0.8	1.8	0.029	0.029	**0.029**	0.029	87.6	0.029	0.029	96.6	0.029	0.029	96.6	0.029	0.029	96.7
1	0.8	2	0.029	0.029	**0.029**	0.029	91.4	0.029	0.029	98.6	0.029	0.029	98.5	0.029	0.029	98.7
1.2	0.2	1.4	0.033	0.033	**0.032**	0.033	26.3	**0.032**	0.033	71.9	**0.032**	0.033	77.8	**0.032**	0.033	78
1.2	0.2	1.6	0.033	0.033	**0.032**	0.033	47	**0.033**	0.033	92.4	**0.033**	0.033	95.3	**0.033**	0.033	95.3
1.2	0.2	1.8	0.033	0.033	**0.033**	0.033	61.6	0.033	0.033	98	0.033	0.033	98.9	0.033	0.033	99
1.2	0.2	2	0.033	0.033	**0.033**	0.033	72.9	0.033	0.033	99.4	0.033	0.033	99.7	0.033	0.033	99.8
1.2	0.4	1.4	0.033	0.033	**0.032**	0.033	26.4	**0.032**	0.033	66.5	**0.032**	0.033	73.1	**0.032**	0.033	72.9
1.2	0.4	1.6	0.033	0.033	**0.032**	0.033	48.1	**0.033**	0.033	90.5	**0.033**	0.033	93	**0.033**	0.033	93.4
1.2	0.4	1.8	0.033	0.033	**0.032**	0.033	62.6	0.033	0.033	96.8	0.033	0.033	97.9	0.033	0.033	98.1
1.2	0.4	2	0.033	0.033	**0.032**	0.033	73.1	0.033	0.033	99.1	0.033	0.033	99.4	0.033	0.033	99.4
1.2	0.6	1.4	0.033	0.033	**0.032**	0.033	27.4	**0.032**	0.033	61.7	**0.032**	0.033	66.9	**0.032**	0.033	66.9
1.2	0.6	1.6	0.033	0.033	**0.032**	0.033	49.5	**0.032**	0.033	88	**0.033**	0.033	90.6	**0.033**	0.033	90.4
1.2	0.6	1.8	0.033	0.033	**0.032**	0.033	64.5	0.033	0.033	95.7	0.033	0.033	96.9	0.033	0.033	97
1.2	0.6	2	0.033	0.033	**0.032**	0.033	75.8	0.033	0.033	98.7	0.033	0.033	99.1	0.033	0.033	99.2
1.2	0.8	1.4	0.033	0.033	**0.032**	0.033	30.6	**0.032**	0.033	56.7	**0.032**	0.033	59.4	**0.032**	0.033	59.8
1.2	0.8	1.6	0.033	0.033	**0.032**	0.033	53.3	**0.032**	0.033	83.9	**0.032**	0.033	86.1	**0.032**	0.033	86.4
1.2	0.8	1.8	0.033	0.033	**0.032**	0.033	69.7	0.033	0.033	94.3	0.033	0.033	94.8	0.033	0.033	95
1.2	0.8	2	0.033	0.033	**0.032**	0.033	81.2	0.033	0.033	97.9	0.033	0.033	98.5	0.033	0.033	98.5
1.2	1	1.4	0.033	0.033	**0.032**	0.033	36	**0.032**	0.033	52.4	**0.032**	0.033	52.7	**0.032**	0.033	52.8
1.2	1	1.6	0.033	0.033	**0.032**	0.033	60.9	**0.032**	0.033	80	**0.032**	0.033	81.2	**0.032**	0.033	81.6
1.2	1	1.8	0.033	0.033	**0.032**	0.033	75.5	0.033	0.033	91.8	0.033	0.033	91.6	0.033	0.033	92.4
1.2	1	2	0.033	0.033	**0.033**	0.033	85.4	0.033	0.033	97.4	0.033	0.033	97.1	0.033	0.033	97.4
1.4	0.2	1.6	0.037	0.037	**0.036**	0.037	22.4	**0.036**	0.037	74.8	**0.036**	0.037	80.3	**0.036**	0.037	80.3
1.4	0.2	1.8	0.037	0.037	**0.036**	0.037	40.5	**0.037**	0.037	91.6	0.037	0.037	94.7	0.037	0.037	94.8
1.4	0.2	2	0.037	0.037	**0.036**	0.037	56.3	0.037	0.037	98.8	0.037	0.037	99.5	0.037	0.037	99.5
1.4	0.4	1.6	0.037	0.037	**0.036**	0.037	23.3	**0.036**	0.037	71.5	**0.036**	0.037	76.6	**0.036**	0.037	76.6
1.4	0.4	1.8	0.037	0.037	**0.036**	0.037	40.8	**0.036**	0.037	89.7	**0.037**	0.037	92.6	**0.037**	0.037	92.7
1.4	0.4	2	0.037	0.037	**0.036**	0.037	57	0.037	0.037	98.4	0.037	0.037	99	0.037	0.037	98.8
1.4	0.6	1.6	0.037	0.037	**0.036**	0.037	24.8	**0.036**	0.037	67.5	**0.036**	0.037	72.9	**0.036**	0.037	72.6

1.4	0.6	1.8	0.037	0.037	**0.036**	0.037	42.9	**0.036**	0.037	86.9	**0.036**	0.037	90.7	**0.036**	0.037	90.4
1.4	0.6	2	0.037	0.037	**0.036**	0.037	59.1	**0.037**	0.037	97.6	0.037	0.037	98.5	0.037	0.037	98.5
1.4	0.8	1.6	0.037	0.037	**0.036**	0.037	26.3	**0.036**	0.037	62	**0.036**	0.037	66.2	**0.036**	0.037	66.2
1.4	0.8	1.8	0.037	0.037	**0.036**	0.037	45	**0.036**	0.037	82.9	**0.036**	0.037	85.3	**0.036**	0.037	85.4
1.4	0.8	2	0.037	0.037	**0.036**	0.037	61.2	**0.036**	0.037	96.1	**0.037**	0.037	97.3	**0.037**	0.037	97.5
1.4	1	1.6	0.037	0.037	**0.036**	0.037	29.8	**0.036**	0.037	56.9	**0.036**	0.037	60.1	**0.036**	0.037	60
1.4	1	1.8	0.037	0.037	**0.036**	0.037	49.7	**0.036**	0.037	80.3	**0.036**	0.037	82.2	**0.036**	0.037	82.6
1.4	1	2	0.037	0.037	**0.036**	0.037	67.3	**0.036**	0.037	95	**0.036**	0.037	95.9	**0.036**	0.037	96.1
1.4	1.2	1.6	0.037	0.037	**0.035**	0.037	35.3	**0.035**	0.037	50.6	**0.035**	0.037	52.5	**0.035**	0.037	52.6
1.4	1.2	1.8	0.037	0.037	**0.036**	0.037	55.4	**0.036**	0.037	74.9	**0.036**	0.037	76.8	**0.036**	0.037	76.7
1.4	1.2	2	0.037	0.037	**0.036**	0.037	73.4	**0.036**	0.037	91.8	**0.036**	0.037	91.9	**0.036**	0.037	92.3
1.6	0.2	1.8	0.041	0.041	**0.040**	0.041	19.9	**0.040**	0.041	75.6	**0.040**	0.041	80	**0.040**	0.041	80
1.6	0.2	2	0.041	0.041	**0.040**	0.041	36.9	0.040	0.041	93.3	0.041	0.041	95.8	0.041	0.041	95.9
1.6	0.4	1.8	0.041	0.041	**0.040**	0.041	19.3	**0.040**	0.041	72.2	**0.040**	0.041	76.5	**0.040**	0.041	76.5
1.6	0.4	2	0.041	0.041	**0.040**	0.041	34.9	0.040	0.041	91.4	0.040	0.041	93.9	0.040	0.041	93.8
1.6	0.6	1.8	0.041	0.041	**0.040**	0.041	18.5	**0.040**	0.041	68.7	**0.040**	0.041	73.6	**0.040**	0.041	73.6
1.6	0.6	2	0.041	0.041	**0.040**	0.041	35	**0.040**	0.041	89.2	**0.040**	0.041	91.6	**0.040**	0.041	91.6
1.6	0.8	1.8	0.041	0.041	**0.040**	0.041	23	**0.040**	0.041	65.5	**0.040**	0.041	69.6	**0.040**	0.041	69.6
1.6	0.8	2	0.041	0.041	**0.040**	0.041	40.7	**0.040**	0.041	88	**0.040**	0.041	90.2	**0.040**	0.041	90.3
1.6	1	1.8	0.041	0.041	**0.040**	0.041	26	**0.040**	0.041	61.5	**0.040**	0.041	65.2	**0.040**	0.041	65.3
1.6	1	2	0.041	0.041	**0.040**	0.041	45.2	**0.040**	0.041	85.2	**0.040**	0.041	87.7	**0.040**	0.041	87.7
1.6	1.2	1.8	0.041	0.041	**0.040**	0.041	29.5	**0.040**	0.041	56.3	**0.040**	0.041	59.1	**0.040**	0.041	59.4
1.6	1.2	2	0.041	0.041	**0.040**	0.041	50.9	**0.040**	0.041	82.1	**0.040**	0.041	84.4	**0.040**	0.041	84.4
1.6	1.4	1.8	0.041	0.041	**0.039**	0.041	33.5	**0.039**	0.041	49.9	**0.039**	0.041	51.1	**0.039**	0.041	51
1.6	1.4	2	0.041	0.041	**0.040**	0.041	57.2	**0.040**	0.041	76.5	**0.040**	0.041	77.4	**0.040**	0.041	77.8
1.8	0.2	2	0.045	0.044	0.044	0.045	14.5	**0.044**	0.045	75.6	**0.044**	0.045	80.9	**0.044**	0.045	81
1.8	0.4	2	0.045	0.045	**0.044**	0.045	14.3	**0.044**	0.045	72.5	**0.044**	0.045	77.9	**0.044**	0.045	77.9
1.8	0.6	2	0.045	0.045	**0.044**	0.045	14.6	**0.044**	0.045	70	**0.044**	0.045	74.9	**0.044**	0.045	75
1.8	0.8	2	0.045	0.045	**0.044**	0.045	16.8	**0.044**	0.045	68.1	**0.044**	0.045	72.5	**0.044**	0.045	72.5
1.8	1	2	0.045	0.044	**0.044**	0.045	20	**0.044**	0.045	64.8	**0.044**	0.045	68.1	**0.044**	0.045	67.9
1.8	1.2	2	0.045	0.045	**0.044**	0.045	24.5	**0.044**	0.045	60.8	**0.044**	0.045	64.2	**0.044**	0.045	64
1.8	1.4	2	0.045	0.045	**0.043**	0.045	29	**0.043**	0.045	56.5	**0.043**	0.045	58.6	**0.043**	0.045	58.8
1.8	1.6	2	0.045	0.045	**0.043**	0.045	31.8	**0.043**	0.045	48.5	**0.043**	0.045	49.5	**0.043**	0.045	49.7

Table S4. d_{XY} between P_2 and P_3 for different sets of windows, for NULL MODELS

The first two columns give the split times for the model in units of 4N generations ago (see Fig. 4D). For each of the introgression statistics, there are two columns giving d_{XY} between P_2 and P_3 for outliers (windows with the top 10% of values), and non-outliers (the remaining 90% of windows). Highlighted blocks indicate cases where d_{XY} in the outlier windows was significantly lower than in the non-outlier windows.

		D		f_d		f_G		f_{hom}	
		outlier	non-outlier	outlier	non-outlier	outlier	non-outlier	outlier	non-outlier
0.4	0.2	**0.017**	0.018	**0.017**	0.018	**0.016**	0.018	**0.017**	0.018
0.6	0.2	**0.021**	0.022	**0.021**	0.022	**0.021**	0.022	**0.021**	0.022
0.6	0.4	**0.020**	0.022	**0.020**	0.022	**0.020**	0.022	**0.020**	0.022
0.8	0.2	**0.025**	0.026	**0.025**	0.026	**0.025**	0.026	**0.025**	0.026
0.8	0.4	**0.025**	0.026	**0.024**	0.026	**0.024**	0.026	**0.024**	0.026
0.8	0.6	**0.024**	0.026	**0.024**	0.026	**0.024**	0.026	**0.024**	0.026
1	0.2	**0.029**	0.029	**0.029**	0.029	**0.029**	0.029	**0.029**	0.029
1	0.4	**0.029**	0.029	**0.029**	0.029	**0.029**	0.029	**0.029**	0.029
1	0.6	**0.028**	0.029	**0.028**	0.029	**0.028**	0.029	**0.028**	0.029
1	0.8	**0.028**	0.030	**0.028**	0.029	**0.028**	0.030	**0.028**	0.030
1.2	0.2	0.033	0.033	**0.033**	0.033	**0.033**	0.033	**0.033**	0.033
1.2	0.4	**0.033**	0.033	**0.033**	0.033	**0.032**	0.033	**0.032**	0.033
1.2	0.6	**0.032**	0.033	**0.032**	0.033	**0.032**	0.033	**0.032**	0.033
1.2	0.8	**0.032**	0.033	**0.032**	0.033	**0.032**	0.033	**0.032**	0.033
1.2	1	**0.032**	0.033	**0.032**	0.033	**0.032**	0.033	**0.032**	0.033
1.4	0.2	0.037	0.037	**0.036**	0.037	**0.036**	0.037	**0.036**	0.037
1.4	0.4	**0.036**	0.037	**0.037**	0.037	**0.036**	0.037	**0.036**	0.037
1.4	0.6	**0.036**	0.037	**0.036**	0.037	**0.036**	0.037	**0.036**	0.037
1.4	0.8	**0.036**	0.037	**0.036**	0.037	**0.036**	0.037	**0.036**	0.037
1.4	1.2	**0.036**	0.037	**0.036**	0.037	**0.036**	0.037	**0.036**	0.037
1.4	1	**0.036**	0.037	**0.036**	0.037	**0.035**	0.037	**0.035**	0.037
1.6	0.2	**0.040**	0.041	**0.040**	0.041	**0.040**	0.041	**0.040**	0.041
1.6	0.4	**0.040**	0.041	**0.040**	0.041	**0.040**	0.041	**0.040**	0.041
1.6	0.6	**0.040**	0.041	**0.040**	0.041	**0.040**	0.041	**0.040**	0.041
1.6	0.8	**0.040**	0.041	**0.040**	0.041	**0.040**	0.041	**0.040**	0.041
1.6	1.2	**0.040**	0.041	**0.040**	0.041	**0.040**	0.041	**0.040**	0.041
1.6	1.4	**0.040**	0.041	**0.040**	0.041	**0.040**	0.041	**0.040**	0.041
1.6	1	**0.039**	0.041	**0.039**	0.041	**0.039**	0.041	**0.039**	0.041
1.8	0.2	0.044	0.044	**0.044**	0.045	**0.044**	0.045	**0.044**	0.045
1.8	0.4	0.044	0.045	0.044	0.045	**0.044**	0.045	0.044	0.045
1.8	0.6	**0.044**	0.045	**0.044**	0.045	**0.044**	0.045	**0.044**	0.045
1.8	0.8	**0.044**	0.045	**0.044**	0.045	**0.044**	0.045	**0.044**	0.045
1.8	1.2	**0.044**	0.045	**0.044**	0.045	**0.044**	0.045	**0.044**	0.045
1.8	1.4	**0.044**	0.045	**0.044**	0.045	**0.044**	0.045	**0.044**	0.045
1.8	1.6	**0.044**	0.045	**0.044**	0.045	**0.043**	0.045	**0.043**	0.045
1.8	1	**0.043**	0.045	**0.043**	0.045	**0.043**	0.045	**0.043**	0.045
2	0.2	0.048	0.048	**0.048**	0.048	**0.048**	0.048	**0.048**	0.048
2	0.4	0.048	0.048	**0.048**	0.048	**0.048**	0.048	**0.048**	0.048
2	0.6	**0.048**	0.048	**0.048**	0.048	**0.048**	0.048	**0.048**	0.048
2	0.8	**0.048**	0.048	**0.048**	0.048	**0.048**	0.048	**0.048**	0.048
2	1.2	**0.048**	0.048	**0.048**	0.048	**0.048**	0.048	**0.048**	0.048

2	1.4	**0.048**	0.048	**0.047**	0.048	**0.047**	0.048	**0.047**	0.048
2	1.6	**0.047**	0.048	**0.047**	0.048	**0.047**	0.048	**0.047**	0.048
2	1.8	**0.047**	0.048	**0.047**	0.048	**0.047**	0.048	**0.047**	0.048
2	1	**0.047**	0.048	**0.047**	0.048	**0.047**	0.048	**0.047**	0.048
0.4	0.2	**0.016**	0.018	**0.016**	0.018	**0.015**	0.018	**0.015**	0.018
0.6	0.2	**0.020**	0.022	**0.020**	0.022	**0.019**	0.022	**0.019**	0.022
0.6	0.4	**0.020**	0.022	**0.019**	0.022	**0.019**	0.022	**0.019**	0.022
0.8	0.2	**0.025**	0.026	**0.024**	0.026	**0.024**	0.026	**0.024**	0.026
0.8	0.4	**0.024**	0.026	**0.023**	0.026	**0.023**	0.026	**0.023**	0.026
0.8	0.6	**0.023**	0.026	**0.023**	0.026	**0.022**	0.026	**0.022**	0.026
1	0.2	**0.029**	0.029	**0.029**	0.029	**0.028**	0.029	**0.028**	0.029
1	0.4	**0.028**	0.029	**0.028**	0.029	**0.028**	0.029	**0.028**	0.029
1	0.6	**0.028**	0.030	**0.027**	0.030	**0.027**	0.030	**0.027**	0.030
1	0.8	**0.027**	0.030	**0.026**	0.030	**0.026**	0.030	**0.026**	0.030
1.2	0.2	0.033	0.033	**0.032**	0.033	**0.032**	0.033	**0.032**	0.033
1.2	0.4	**0.033**	0.033	**0.032**	0.033	**0.032**	0.033	**0.032**	0.033
1.2	0.6	**0.032**	0.033	**0.032**	0.033	**0.031**	0.033	**0.031**	0.033
1.2	0.8	**0.031**	0.033	**0.031**	0.033	**0.031**	0.034	**0.031**	0.034
1.2	1	**0.031**	0.034	**0.030**	0.034	**0.030**	0.034	**0.030**	0.034
1.4	0.2	0.037	0.037	**0.036**	0.037	**0.036**	0.037	**0.036**	0.037
1.4	0.4	0.036	0.037	**0.036**	0.037	**0.035**	0.037	**0.036**	0.037
1.4	0.6	**0.036**	0.037	**0.036**	0.037	**0.035**	0.037	**0.035**	0.037
1.4	0.8	**0.036**	0.037	**0.035**	0.037	**0.035**	0.037	**0.035**	0.037
1.4	1.2	**0.035**	0.037	**0.035**	0.037	**0.035**	0.037	**0.035**	0.037
1.4	1	**0.034**	0.037	**0.034**	0.037	**0.034**	0.037	**0.034**	0.037
1.6	0.2	0.040	0.041	**0.040**	0.041	**0.040**	0.041	**0.040**	0.041
1.6	0.4	0.041	0.041	**0.040**	0.041	**0.039**	0.041	**0.039**	0.041
1.6	0.6	0.040	0.041	**0.040**	0.041	**0.039**	0.041	**0.040**	0.041
1.6	0.8	**0.040**	0.041	**0.039**	0.041	**0.039**	0.041	**0.039**	0.041
1.6	1.2	**0.039**	0.041	**0.039**	0.041	**0.039**	0.041	**0.039**	0.041
1.6	1.4	**0.039**	0.041	**0.039**	0.041	**0.038**	0.041	**0.039**	0.041
1.6	1	**0.038**	0.041	**0.038**	0.041	**0.038**	0.041	**0.038**	0.041
1.8	0.2	**0.044**	0.045	**0.043**	0.045	**0.043**	0.045	**0.043**	0.045
1.8	0.4	0.044	0.045	**0.043**	0.045	**0.043**	0.045	**0.043**	0.045
1.8	0.6	0.044	0.045	**0.043**	0.045	**0.043**	0.045	**0.043**	0.045
1.8	0.8	0.044	0.045	**0.043**	0.045	**0.043**	0.045	**0.043**	0.045
1.8	1.2	**0.044**	0.045	**0.043**	0.045	**0.043**	0.045	**0.043**	0.045
1.8	1.4	**0.044**	0.045	**0.043**	0.045	**0.043**	0.045	**0.043**	0.045
1.8	1.6	**0.043**	0.045	**0.042**	0.045	**0.042**	0.045	**0.042**	0.045
1.8	1	**0.042**	0.045	**0.042**	0.045	**0.041**	0.045	**0.042**	0.045
2	0.2	0.048	0.048	**0.047**	0.049	**0.047**	0.049	**0.047**	0.049
2	0.4	0.048	0.048	**0.047**	0.048	**0.047**	0.048	**0.047**	0.048
2	0.6	0.048	0.048	**0.047**	0.048	**0.047**	0.048	**0.047**	0.048
2	0.8	**0.048**	0.048	**0.047**	0.048	**0.047**	0.048	**0.047**	0.048
2	1.2	0.048	0.048	**0.047**	0.048	**0.047**	0.049	**0.047**	0.049
2	1.4	**0.047**	0.048	**0.047**	0.049	**0.047**	0.049	**0.047**	0.049
2	1.6	**0.048**	0.048	**0.047**	0.049	**0.047**	0.049	**0.047**	0.049
2	1.8	**0.047**	0.049	**0.046**	0.049	**0.046**	0.049	**0.046**	0.049
2	1	**0.046**	0.048	**0.045**	0.048	**0.045**	0.048	**0.045**	0.048
